# Pharmacological evaluation of drug therapies in Aicardi-Goutières syndrome: insights from patient-derived neural stem cells

**DOI:** 10.3389/fphar.2025.1549183

**Published:** 2025-03-20

**Authors:** Stefania Braidotti, Rosalba Monica Ferraro, Raffaella Franca, Elena Genova, Francesco Giambuzzi, Andrea Mancini, Valentina Marinozzi, Letizia Pugnetti, Giulia Zudeh, Alessandra Tesser, Alberto Tommasini, Giuliana Decorti, Silvia Clara Giliani, Gabriele Stocco

**Affiliations:** ^1^ Department of Paediatrics, Institute for Maternal and Child Health (I.R.C.C.S) Burlo Garofolo, Trieste, Italy; ^2^ “Angelo Nocivelli” Institute for Molecular Medicine, ASST Spedali Civili, Brescia, Italy; ^3^ Department of Molecular and Translational Medicine, University of Brescia, Brescia, Italy; ^4^ Department of Medical, Surgical and Health Sciences, University of Trieste, Trieste, Italy; ^5^ Department of Advanced Translational Diagnostics, Institute for Maternal & Child Health (I.R.C.C.S) Burlo Garofolo, Trieste, Italy

**Keywords:** patient-derived stem cell, Aicardi-Goutières syndrome, drug sensitivity, JAK inhibitors, antiretrovirals

## Abstract

Aicardi-Goutières syndrome (AGS) is a rare genetic disorder classified among type I interferonopathies. Current pharmacological management of AGS is symptomatic and supportive, with recent clinical applications of JAK inhibitors (JAKi) and antiretroviral therapies (RTIs). To investigate the effects of these therapies, patient-specific induced pluripotent stem cells (iPSCs) were generated by reprogramming fibroblasts from three AGS patients with distinct genetic mutations (AGS1, AGS2, AGS7) and differentiated into neural stem cells (NSCs). iPSCs and NSCs derived from commercial BJ fibroblasts of a healthy donor served as control. The cytotoxic effects of glucocorticoids, thiopurines, JAK inhibitors (ruxolitinib, baricitinib, tofacitinib, pacritinib), and RTIs (abacavir, lamivudine, zidovudine) were evaluated using the MTT assay. Results showed that glucocorticoids did not compromise NSC viability. Among thiopurines, thioguanine, but not mercaptopurine, exhibited cytotoxicity in NSCs. All tested JAK inhibitors, except pacritinib, were non-toxic to iPSCs and NSCs. Interestingly, high concentrations of certain JAK inhibitors (ruxolitinib, baricitinib, tofacitinib) led to an unexpected increase in cell viability in AGS patient-derived cells compared to control, suggesting potential alterations in cell proliferation or stress responses. RTIs demonstrated no cytotoxicity, except for zidovudine, which showed selective toxicity in AGS2-derived iPSCs compared to controls. These findings suggest that glucocorticoids, JAK inhibitors (excluding pacritinib), and RTIs are likely safe for NSCs of AGS patients, while caution is warranted with thioguanine and pacritinib. Further studies are needed to explore the mechanisms underlying increased cell viability at high JAK inhibitor concentrations and the selective sensitivity to zidovudine.

## 1 Introduction

Aicardi-Goutieres syndrome (AGS, ORPHA-51 (www.orpha.net)) is a rare genetic neurological disorder classified as type I interferonopathy (Landrieu et al., 2013). It typically begins during infancy and early childhood. The prevalence is about 1–5 cases every 10,000 persons and the disease affects individuals across various ethnic backgrounds. AGS was first described in 1984 in patients showing early-onset encephalopathy, basal ganglia calcification and persistent lymphocytosis in cerebro-spinal fluid (CSF) causing systemic inflammation and neurological progressive impairments with poor life expectancy (Aicardi and Goutières, 1984; Lebon et al., 1988). Tetraparesis occurs without other obvious causes in the first year of life and most of the affected children exhibit intellectual disability, dystonia, microcephaly and skin lesions similar to chilblains at fingers and toes (Al Mutairi et al., 2018). The main neuropathological feature of the disease is abnormal myelination, likely caused by increased expression of proteases like cathepsin D, which can degrade myelin and brain tissue matrix (Pulliero et al., 2013; Vanderver et al., 2015). Elevated type I interferons (IFNs) in CSF and serum are additional hallmarks typically associated to AGS (La Piana et al., 2016; Al Mutairi et al., 2018). AGS patients show an increased expression of interferon-stimulated genes (ISGs), the so-called “interferon signature”, commonly observed in CD4^+^ T cells, monocytes, and monocyte-derived macrophages from peripheral blood (Rice et al., 2013; Galli et al., 2018; Aso et al., 2019).

To date, nine subtypes of AGS have been identified, each associated with mutations in different genes (i.e., DNA exonuclease 1 (*TREX1*) in AGS1, ribonuclease H2 subunit B (*RNASEH2B*) in AGS2, ribonuclease H2 subunit C (*RNASEH2C*) in AGS3, ribonuclease H2 subunit A (*RNASEH2A*) in AGS4, SAM and HD domain containing deoxynucleoside triphosphate triphosphohydrolase 1 (*SAMHD1*) in AGS5, RNA-specific adenosine deaminase-1 (*ADAR1*) in AGS6, cytosolic double-stranded RNA receptor gene *IFIH1* (also called MDA5) in AGS7, U7 Small Nuclear RNA Associated (*LSM11*) in AGS8 and U7 Small Nuclear 1 (*RNU7-1*) in AGS9 (Crow et al., 2015; Livingston and Crow, 2016; Uggenti et al., 2020)). These genes encode proteins involved in nucleotide metabolism and/or sensing. Consequently, AGS is associated with an abnormal response to endogenous nucleic acid stimuli. Specifically, the accumulation of cytosolic DNA activates the DNA sensor cyclic GMP-AMP synthase (cGAS). Upon binding to an endogenous nucleic acid fragment, cGAS converts AMP and GMP into cyclic 2′3′GMP-AMP (cGAMP) that acts as a second messenger by interacting with STimulator of INterferon Genes (STING) localized on the endoplasmic reticulum (Sun et al., 2013). STING then translocates from the endoplasmic reticulum to the Golgi, where it recruits the TBK1 kinase and subsequently the IFN regulatory factor IRF(3), which translocates to the nucleus to upregulate the production and the release of type I IFNs.

The transcriptional induction of the genes encoding type I IFNs is achieved also by the activation of cellular sensors for RNA accumulation localized in the cytosol. Indeed, gain-of-function mutations in the *IFIH1* gene encoding MDA5 (AGS7) activate the retinoic acid-inducible gene I (RIG-I)-like receptors (RLRs) pathway rather than the cGAS-STING pathway. MDA5 associates with the adapter molecule mitochondrial antiviral-signaling protein (MAVS). This interaction leads to the recruitment of downstream signaling molecules, including TNF receptor associated factor (TRAF) 3/6 and inhibitor of NF-κB kinase (IKK) family members (IKKε, TBK1, and IKKα/β), to activate IRF-3/7 and NF-κB, leading to the transcriptional activation of IFN and proinflammatory cytokine genes (Onomoto et al., 2021). Mutations in the *ADAR1* gene encoding the adenosine deaminase acting on RNA 1 (AGS6) impairs the adenosine to inosine conversion in double-stranded RNA, disrupting a RNA editing process (Rehwinkel and Mehdipour, 2024). Loss of function mutations in *ADAR1* lead to recognition of unedited self-dsRNAs by MDA5, which activates MAVS, TBK1 and IRF3 in turn, thus inducing overexpression of type I IFNs (Liu and Ying, 2023).

Type I IFNs act in autocrine and paracrine manner. When type I IFNs bind to their cell surface receptors, IFNAR1 and IFNAR2, the receptor-associated tyrosine kinases JAK1 and TYK2 are activated and phosphorylate the receptors themselves, creating docking sites for STAT1 and STAT2 proteins that are in turn recruited to the receptor and phosphorylated. This activation step further promotes the dimerization of STAT1 and STAT2 as well as the recruitment of IRF9 to form the heterotrimeric interferon-stimulated gene factor 3 (ISGF3) transcription complex. In the nucleus, ISGF3 binds to IFN-stimulated response elements (ISRE) on DNA, promoting the expression of ISGs (Schneider et al., 2014; Volpi et al., 2016; Michalska et al., 2018; Fryer et al., 2021).

To date, there are no effective cures for AGS patients. In the past, treatments were mainly supportive and symptomatic, aimed at improving the patient’s quality of life particularly if started early, during the initial acute/subacute phase of the disease. “Broad acting” immunosuppressive therapies, based on glucocorticoids (dexamethasone, methylprednisolone), intravenous immunoglobulin (IVIG) and thiopurines (mercaptopurine, thioguanine and azathioprine) and/or combined (prednisone + azathioprine, intravenous methylprednisolone + IVIG) were tried empirically for symptomatic relief through the manage and reduction the inflammatory conditions (Crow, 2003; Crow et al., 2014; Iro et al., 2017; Tonduti et al., 2020). Evaluating efficacy of these interventions was challenging because of the small number of patients involved and variability in the stage of the disease process at which treatment was initiated.

Insight into the pathogenic mechanism of AGS have suggested novel therapeutic strategies, including the use of JAK inhibitors (JAKi) and reverse transcriptase inhibitors (RTIs), (Tonduti et al., 2020; Cattalini et al., 2021; Galli et al., 2023). Since JAK1 and TYK2 play a pivotal role in IFNAR1 and IFNAR2 activation and in type I IFNs response, JAKi are considered promising in reducing the inflammation caused by the IFNs overproduction (Tonduti, et al., 2020; Vanderver et al., 2020); current ongoing clinical trials are exploring the use of baricitinib (a JAK1/JAK2 non selective inhibitor) in AGS and AGS-related interferonopathies (NCT03921554, NCT04517253, clinicaltrials.gov). Another current hypothesis is that functional loss of an AGS-associated gene alters the normal metabolism of retrotransposon RNA or its reverse-transcribed cDNA, triggering an interferon response and AGS. RTI can potentially disrupt the replication cycle of both exogenous retroviruses and endogenous retro-elements, reducing the accumulation of cytosolic DNA, which is the responsible for IFN release (Crow et al., 2014). Using a combination of three RTI (abacavir, lamivudine and zidovudine), Rice and collaborators demonstrated a reduction in IFN signaling without evidence of side effects, leading to a reduction in IFN score, calculated as the median fold change of the six target ISGs (Rice et al., 2013). The clinical trial NCT04731103 assessed the interferon status in AGS patients. NCT03304717 investigates whether RTI can decrease endogenous retroelement accumulation. Novel approaches such as anti-IFN-α antisense oligonucleotides (Viengkhou et al., 2024a), cGAS inhibitors (e.g., antimalarial drugs (mepacrine) (Lama et al., 2019)) and STING inhibitors (Zhang et al., 2023) are currently under investigation but their efficacy still needs to be proven in AGS patients. Unlike prior “broad acting” immunosuppressive therapies that focus on symptoms management, JAKi, RTIs and these latter potential drugs aim to target specifically the underlying pathogenic mechanism of AGS, potentially improving the clinical outcomes of patients.

Given the neurological localization of the disease and the challenges in creating *in vivo* models, the ability to generate innovative patient-specific models, such as neural stem cells (NSCs) derived from induced pluripotent stem cells (iPSCs), offers an intriguing and promising approach for pharmacological studies. The roles of NSC go beyond brain development and are complex and multifaceted. NSC are now recognized for their potential in immunomodulation and in slowing down disease progression in many neurological disorders, although not in the specific context of AGS (Rahimi Darehbagh et al., 2024). We aim to explore whether drugs used in AGS treatment might harm NSCs, which may have beneficial protective functions that have not yet been fully investigated, or ruled out.

Aim of the study was therefore to provide currently missing evidences on JAKi and RTI effects on AGS specific stem cells, chosen as patient-specific and innovative preclinical models; in particular safety of therapy was assessed by drug cytotoxicity on iPSC and NSC. Glucocorticoids and thiopurines were also included in the panel of drugs tested.

## 2 Materials and methods

### 2.1 Drug and chemicals

Ruxolitinib (Catalog Number: 11,609, Cayman Chemical, United States), baricitinib (S2851, Selleckchem, United States), tofacitinib (S5001, Selleckchem, United States), pacritinib (SB1518, Selleck Chemicals, United States), lamivudine (L1295, Sigma-Aldrich, Italy), abacavir sulfate (SML0089, Sigma-Aldrich, Italy), zidovudine (A2169, Sigma-Aldrich, Italy), mercaptopurine (852,678, Sigma-Aldrich, Italy), thioguanine (A4882, Sigma-Aldrich, Italy) were dissolved according to manufacturer instructions (Supplementary Table S1).

### 2.2 iPSC reprogramming

iPSCs were generated and characterized at the “A. Nocivelli” Institute of Molecular Medicine in Brescia (Italy). AGS1-iPSCs were generated from skin fibroblasts of a 5-year-old male with a compound heterozygous *TREX1* mutation (c.[260insAG]; [290G>A]) (Ferraro et al., 2019a). AGS2-iPSCs were generated from skin fibroblasts of a 10-year-old female presenting a homozygous mutation in *RNASEH2B* (c. [529G>A]; [529G>A]) (Ferraro et al., 2019b). AGS7-iPSCs were generated from fibroblasts of a 14-year-old male with a dominant negative heterozygous mutation in *IFIH1* (c.[2471G>A]; wt) (Masneri et al., 2019). Reprogramming was performed in feeder free condition using an episomal Sendai virus-based vector delivering reprogramming factors *OCT4, SOX2, KLF4* and *c-MYC* (CytoTune-iPS 2.0 Sendai Reprogramming Kit, A16517, ThermoFisher Scientific, Italy). IPSCs reprogrammed from the commercial cell line BJ (human foreskin fibroblasts from a neonatal male, ATCC CRL-2522, American Type Culture Collection, Manassas, VA) were used as the control cell line (Ali et al., 2020). The study was approved by the Scientific Committee and by the Board of the Aziende Socio Sanitarie Territoriale Spedali Civili of Brescia, protocol numbers 1603 (AGS-CARIPLO study) and 3426 (iPSCREP study) and appropriate informed consent was obtained from patients’ parents. The study (RC 9/24) was also approved by the Institutional Review Board of IRCCS Burlo Garofolo in Trieste (Italy).

### 2.3 iPSC-derived NSC

For NSCs differentiation, iPSCs colonies were seeded in StemMACS iPS-Brew XF medium on diluted Geltrex coated 6-wells plates at a density of 3 × 10^5^ cells/well and allowed to recover for 24 h at 37°C 5% CO_2_, 20% O_2_. Then, the medium was change to PSC Neural Induction medium (A1647801, Gibco, ThermoFisher Scintific, Italy) and, for 7 days, medium was replaced every other day, according to standard protocol (ThermoFisher Scientific, MAN0008031).

### 2.4 Cell cultures

Patient-specific iPSCs and the control BJ-iPSCs were maintained in StemMACS iPS-Brew XF (130-104-368, Miltenyi Biotec, Italy) on diluted Geltrex matrix (A1413202, Gibco, ThermoFisher Scientific, Italy) coated plates (1 mL/well in 6-well plate, 833,920 Sarstedt, Italy). Geltrex was diluted at a ratio of 1:100 in DMEM/F12 medium (D8062, Sigma-Aldrich, Italy). Cells were passaged at 80% confluence: after 2 min exposure to Versene (0.48 mM, 15040066, Gibco, ThermoFisher Scientific, Italy) cells were gently detached using medium. Since iPSCs grow in clusters, the standard protocol of passaging used for long-term iPSCs cultures avoids the complete breakup of clusters. In contrast, when single-cell culturing was required to perform cytotoxicity assays, iPSCs were treated according to the same procedure but were exposed to Versene for 5–6 min. After each seeding, 10 μM Rho-associated, coiled-coil containing protein kinase (ROCK) inhibitor (Y-27632, 130-103-922, Miltenyi Biotec, Italy) was added to the medium for 24 h to facilitate cells adhesion.

Patient-specific NSCs and the control BJ-NSCs were maintained in a medium consisting of equal parts of PSC Neural Induction Medium and Advanced DMEM/F12, on diluted Geltrex matrix coated plates (1:100 Geltrex in DMEM/F12 medium). Cells were passaged at 80% confluence: after 5 min exposure to StemPro Accutase Cell Dissociation Reagent (A11105, Gibco, ThermoFisher Scientific, Italy) at 37°C, cells were gently detached with PBS (D8537, Sigma-Aldrich, Italy) and filtered with a 100 μm cell strainer (08-771-19, FisherScientific, Italy) before seeding on diluted Geltrex matrix coated plates.

Immortalized lymphoblastoid cell line NALM6 (ACC 128) purchased from the DSMZ GmbH (Germany) was included as positive control when dexamethasone was tested. The cell line grow as single cells in suspension within sterile polystyrene flasks with growth surfaces of 25 cm^2^ or 75 cm^2^, equipped with a filter cap to allow for gas exchange (83.3910/83.3911, Sarstedt, Italy). Cell passaging is performed every 3 or 4 days, under sterile conditions, using RPMI-1640 culture medium (ECB9006L, Euroclone, Italy) supplemented with 1% L-glutamine (ECB3000D-20, Euroclone, Italy), 1% penicillin/streptomycin (FBS P0781, Sigma-Aldrich, Italy), and 10% fetal bovine serum (FBS, F7524, Sigma-Aldrich, Italy). Cell seeding density varies depending on the cell type, ranges from 0.5 to 2 × 10^6^ cells/mL.

#### 2.5 iPSC cell proliferation assay

Cell proliferation was determined by labeling metabolically active iPSCs cells with [3H]-thymidine (NET027X00 1MC, PerkinElmer, Italy). Cells were seeded on a Geltrex coated 96-well plate at different densities (500, 1,000, 2,500, 5,000, 10,000, 30,000 cells/well) and cultured in StemMACS iPS-Brew XF medium for 96 h, with [3H]-thymidine (2.5 μCi/mL) added during the last 5 h. Cells were then washed with PBS, collected, and the radioactivity of the samples was determined by a liquid scintillation analyzer (Wallac 1,450 Microbeta liquid scintillation counter, PerkinElmer, Italy). Count per minute (CPM) data were analyzed.

### 2.6 iPSC cell cycle analysis

Cell cycle of patient-derived AGS/BJ-iPSCs were analyzed by flow-cytometry using the propidium iodide (P4170, Sigma-Aldrich, Italy) cellular uptake assay (Cattalini et al., 2021). Two million cells were fixed in 70% ethanol on ice, washed twice with PBS, and kept in PBS for 1 h at 4°C. Cells were stained overnight with 2 mL of a PBS/EDTA 0.5 mM solution containing 200 µL of propidium iodide (0.1 mg/mL, P4170, Sigma-Aldrich, Italy) and 25 µL of 1 mg/mL RNase (R4875, Sigma-Aldrich, Italy). Samples were analyzed by flow cytometry (CYTOMICSTM FC500, Beckman Coulter Inc. Fullerton, CA) with FCS Express V3 software.

### 2.7 MTT assay

Viability of iPSCs and NSCs was measured after 72 h growth at different cell seeding density (range: 5.0 × 10^3^–3.0 × 10^4^ cells/well on 100 µL/well of Geltrex coated 96-well plate) by using the 3-(4,5-dimethylthiazol-2-yl)- 2,5-diphenyltetrazolium bromide assay (MTT, M2128-1G, Sigma-Aldrich, Italy).

The cytotoxic effect of drugs on iPSCs and NSCs was also determined by MTT assay. iPSCs and NSCs were cultured on a Geltrex coated 96-well plate at different cell densities according to their different cell growth rate, to obtain similar cell densities at 72 h. AGS2 patient-derived iPSCs were seeded at 3.0 × 10^4^ cells/well in a final volume of 100 µL while all the other iPSCs tested (AGS1, AGS7 and BJ-iPSCs) were seeded at 1.0 × 10^4^ cells/well; all NSCs were seeded at 1.0 × 10^4^ cells/well. After 24 h of incubation at 37°C, 5%CO_2_, 20% O_2_, the cultural medium was replaced with a drug-containing medium at serially diluted concentrations (Supplementary Table S1). Experiments were repeated at least three times, and each experimental condition was tested in triplicate. Treated cells were incubated for 72 h at 37°C, adding MTT in each well (0.5 mg/mL) 4 hours before the end of the incubation; cells were then lysed with 100 µL dimethyl sulfoxide (DMSO, 472,301, Sigma-Aldrich, Italy). Absorbance was measured at 540–630 nm wavelength (Fluorostar Omega, BMG, Labtech Germany).

Cell metabolic activity was calculated as % cell activity = (absorbance treated/absorbance control)*100, and considered as a measurement of cell viability. Percentages were graphically reported as a function of the drug concentrations used, expressed in molarity and reported in a logarithmic scale (Log10), obtaining a sigmoidal dose-response curve.

### 2.8 Real-time PCR analysis

Total RNA was extracted with TRIzol reagent (15596018, Invitrogen, ThermoFisher Scientific, Italy) and PureLink RNA mini KIT (12183018A, ThermoFisher Scientific, Italy) according to the manufacturer’s instructions, and quantified using Nanodrop 2000 spectrophotometer (ThermoFisher Scientific, Italy). RNA was reversed-transcribed into cDNA using the High-Capacity RNA-to-cDNA kit (4387406, Applied Biosystem, ThermoFisher Scientific, Italy).

iPSCs and NSCs stemness determination and the analysis of genes involved in drug pathways were performed by real-time PCR using 12.5 ng of cDNA and the KiCqStart SYBR Green qPCR Ready Mix (KCQS00, Sigma-Aldrich, Italy), in a Thermal Cycle Dice Real Time System (BIO-RAD, Italy), using pre-designed primers sequence (KSPQ12012, Sigma-Aldrich, Italy), shown in Supplementary Table S2. Relative quantification is represented as 2^−ΔCt^ with respect to the housekeeping gene beta-actin. All experiments were carried out in technical triplicate and the reproducibility of the observations was confirmed in at least two biological independent experiments.

### 2.9 Statistical analysis

Graphical representation and statistical analysis were performed using the GraphPad Prism 9.4.1 program (GraphPad Software Inc.). All experiments were replicated at least 3 times.

Data obtained from the MTT assay and proliferation assay are presented as mean ± standard error (SE); results were analyzed using a nonlinear regression and half maximal effective concentration (IC_50_) was determined from the dose-response curve. Two-way analysis of variance (ANOVA) followed by Bonferroni’s post-test was used to compare the cell viability percentage of AGS- versus the cell viability percentage of control cells BJ.

All the gene expression analyses through Real-Time PCR were carried out in technical triplicate and the reproducibility of the observations was confirmed in at least two biological independent experiments. Real-time PCR data of relative expression of stemness genes and genes involved in drug pharmacodynamics were analyzed by t-test analysis in iPSCs versus related NSCs, considering the different degree of differentiation as independent variable.

Statistical significance was set at P < 0.05 for all the statistical analyses.

## 3 Results

### 3.1 Characterization of iPSCs and NSCs

#### 3.1.1 Cells stemness evaluation

**FIGURE 1:**
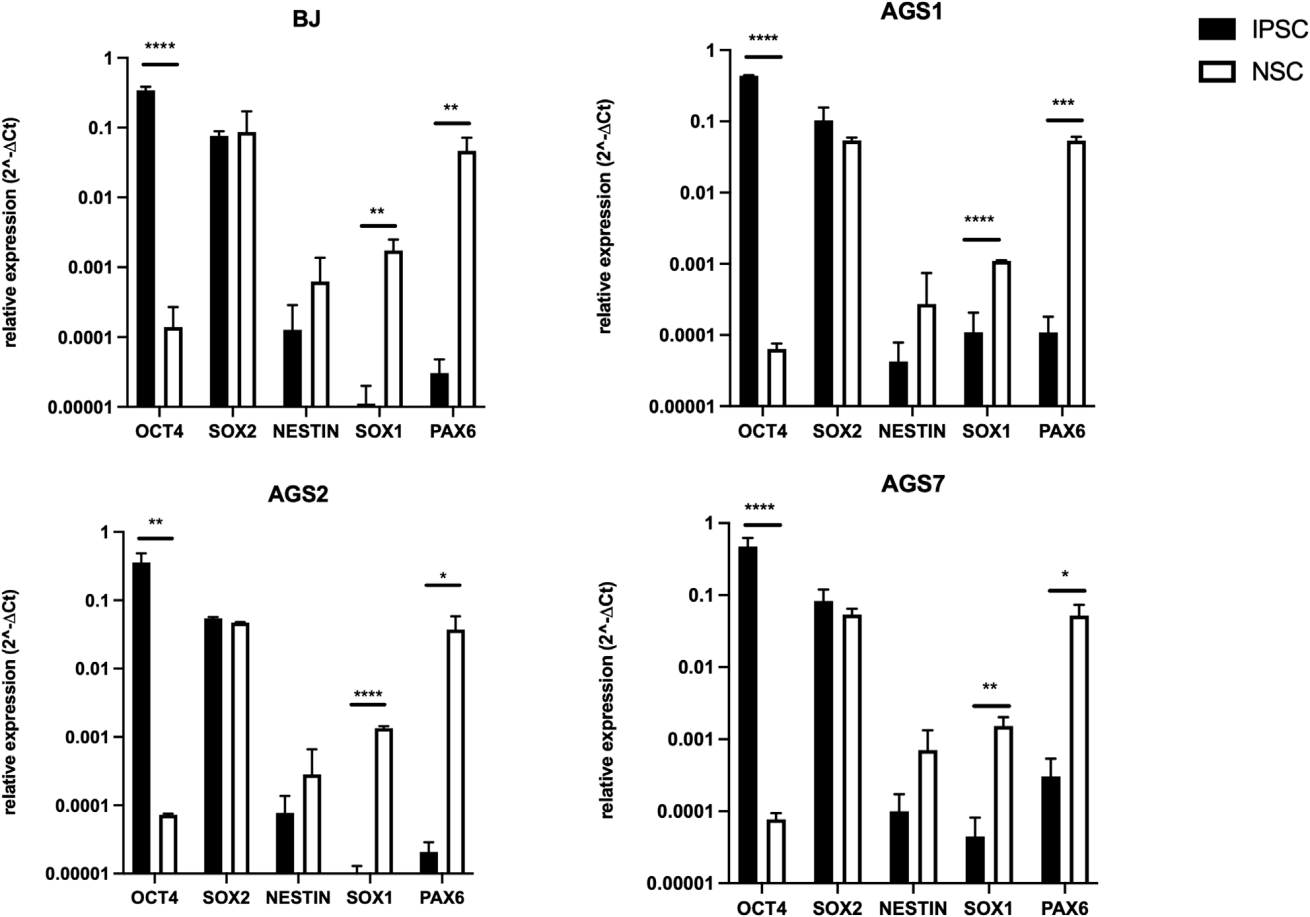
Expression levels of iPSCs (*OCT4, SOX2*) and NSCs stemness specific markers (*SOX2, Nestin, SOX1, PAX6*) in iPSC and NSCs. Gene expression was normalized to housekeeping *actin β* gene expression, and relative expression was calculated as 2^−ΔCt^. P-value according to t-test analysis, *P < 0.05, **P < 0.01, ***P < 0.001, ****P < 0.0001. The data are reported as means ± standard error (SE) of 3 independent experiments performed in triplicate.

Expression of pluripotency gene markers for different grades of stemness was assessed in both AGS patient-derived iPSCs and NSCs by real-time PCR to confirm the proper differentiation of iPSCs into NSCs. The expression of *OCT4* was significantly reduced in NSCs compared to iPSCs whereas the expression of neural markers *SOX1* and *PAX6* were increased. There were no significant variations in *SOX2* and *Nestin* mRNA expression between iPSCs and NSCs in all cell lines considered, although a trend towards an increased *Nestin* mRNA level could be observed in NSC (Figure 1).

#### 3.1.2 Stem cells proliferation and metabolic activity

**FIGURE 2:**
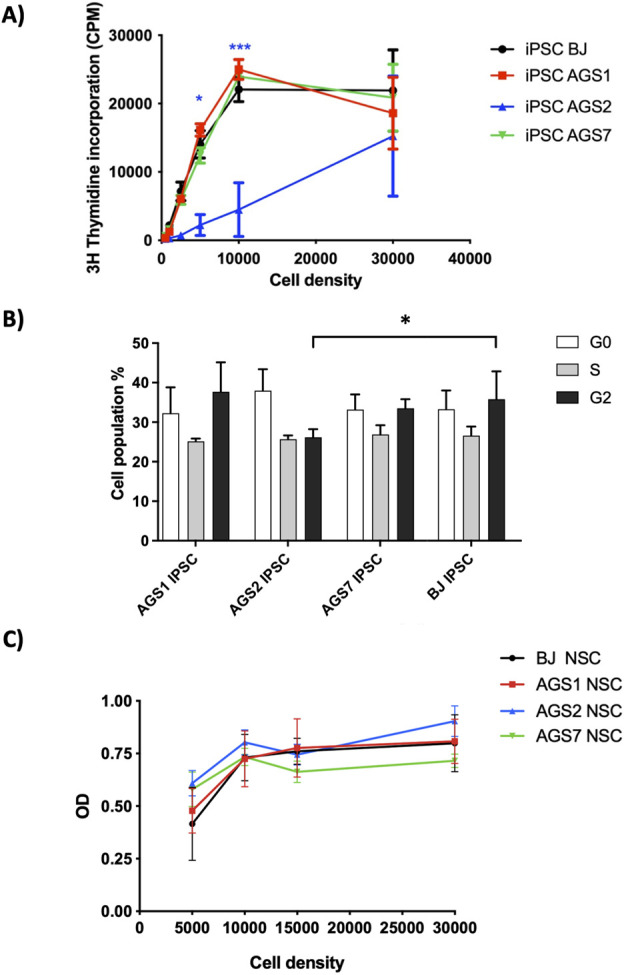
Stem cell viability assays. **(A)** iPSCs proliferation assay; [3H]-thymidine incorporation into DNA was expressed counts per minute (or CPM). **(B)** iPSCs cell cycle assay. **(C)** NSCs MTT assay; NSCs metabolic activity was evaluated under basal conditions. Cell densities referred to initial cell/well seeding in a 96-wells plate. OD: optical density. Data are reported as means ± SE of at least 3 in-dependent experiments performed in triplicate. Statistical analysis vs control BJ-stem cells: *P < 0.05, ***P < 0.001, Two-way ANOVA, and Bonferroni’s post-test.

iPSCs proliferation was analyzed by the [3H]-thymidine incorporation assay and propidium iodide uptake was measured by flow cytometry to analyze iPSCs cell cycle after 72 h of cell culture. AGS2-iPSCs were significantly less proliferating than BJ-iPSCs, particularly when seeded at 0.5 × 10^4^ and 1.0 × 10^4^ cell/well in 96 well plate (Figure 2A). AGS2-iPSCs showed also significantly lower percentage of cells in G2 phase compared to BJ-iPSCs (26.2% vs. 35.8%); in contrast, AGS1-iPSCs and AGS7-iPSCs were comparable to BJ-iPSCs at any phase (Figure 2B).

No difference in metabolic activity, measured by MTT assay, under basal condition was reported for NSCs (Figure 2C). A seeding concentration of 1.0 × 10^4^ NSCs/well was considered the best seeding condition to reach 80%–90% confluence at the end of the 72 h incubation for investigating drug safety.

### 3.2 Drug safety: *in vitro* drugs cytotoxicity on stem cells and analysis of key genes involved in drug pathways

Drug cytotoxic after 72 h of exposure on iPSCs and on NSCs was investigated *in vitro* by MTT assay. The expression of crucial molecular targets involved in drug pharmacodynamic was also evaluated.

#### 3.2.1 Immunosuppressant drugs: glucocorticoids and thiopurines

**FIGURE 3:**
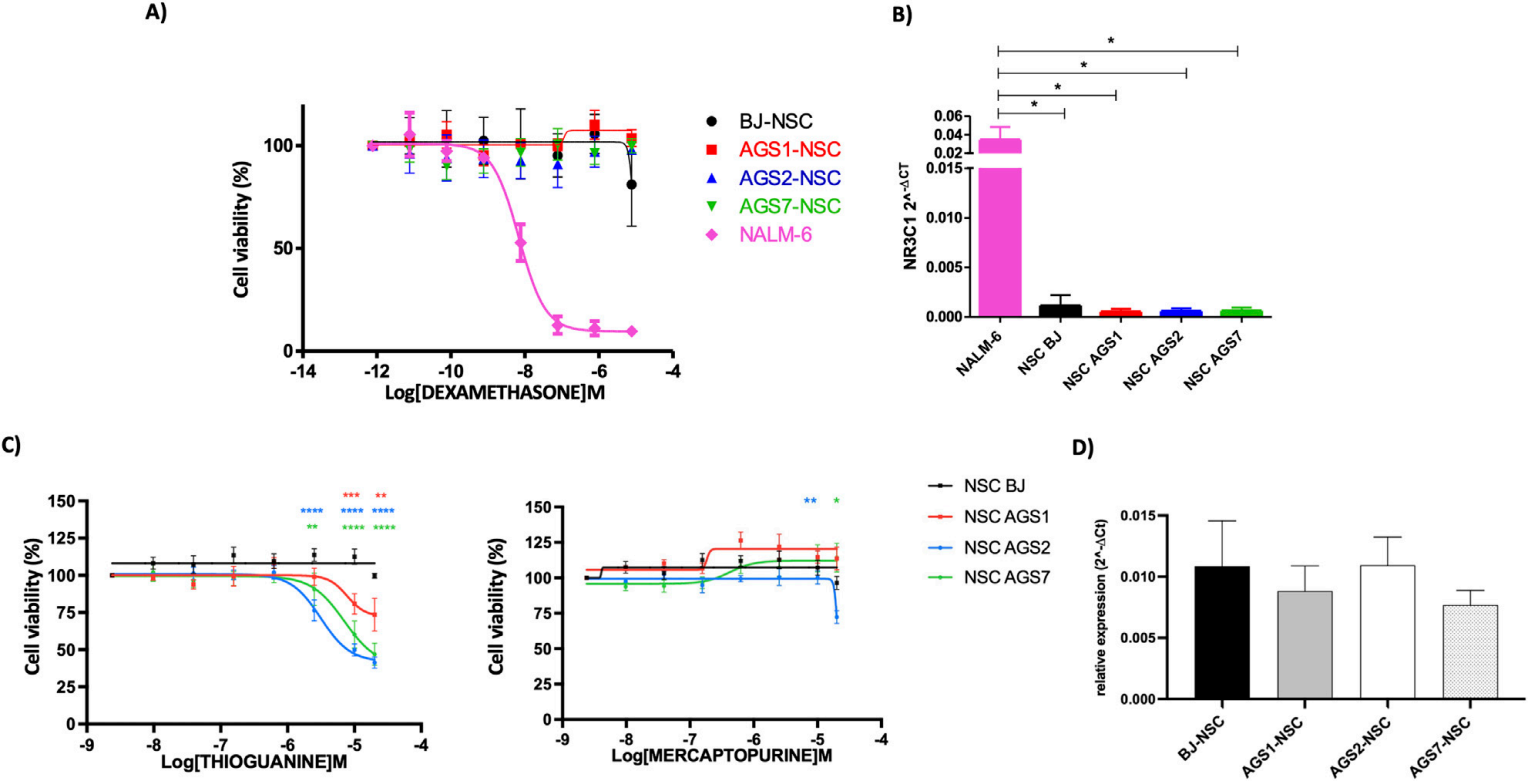
Drug safety of immunosuppressant drugs. **(A)** Dose-response curve (by MTT assay) of dexamethasone on NSCs. Immortalized lymphoblastoid cell line NALM6 was used as control. **(B)** Relative *NR3C1* gene expression. **(C)** Dose-response curve (by MTT assay) of thiopurines on NSCs. **(D)** Relative *HPRT1* gene expression. Drug cytotoxic effects were analyzed by MTT assay after 72 h; results are presented as mean ± standard error (SE) from at least 3 independent experiments for each cell line; P-value were calculated according to Two-way ANOVA, Bonferroni’s post-test. Gene expression was calculated with respect the housekeeping gene *actin β* (relative expression of ΔCT (2^−ΔCT^)); P-value were calculated according to one-way ANOVA analysis. *, p < 0.05, **, p < 0.01, ***, p < 0.001,****, p < 0.0001.

Drug sensitivity of iPSCs to glucocorticoids and thiopurines was previously reported (Genova et al., 2020); NSCs results are reported in Figure 3. Dexamethasone was not cytotoxic for NSCs (Figure 3A). Expression of *NR3C1* gene, encoding the glucocorticoid intracellular receptor as crucial molecular target involved in the glucocorticoid response, was analyzed to understand whether the lack of *in vitro* drug cytotoxicity could be ascribable to the lack of expression of the glucocorticoid intracellular receptor. Indeed, *NR3C1* was not expressed in NSC (Figure 3B).

BJ-NSCs did not show cytotoxicity after thioguanine exposure, whereas a dose-dependent decrease in cell survival was detectable in AGS-derived NSCs (Figure 3C). In AGS2-NSCs and AGS7-NSCs, IC_50_ (mean ± SE) were 4.5 ± 2.8 μM and 14.1 ± 4.4 μM, respectively; however the viability of AGS1-NSCs at 20 μM was 74%, thus IC_50_ could not be calculated. NSCs did not show cytotoxicity also to mercaptopurine. Only a slight cytotoxic effect was observed for AGS2-NSC at the highest concentration tested (viability ∼80%, IC_50_ could not be calculated). HPRT1 is the transferase required for the thiopurine conversion into active metabolites. The expression of *HPRT1,* key gene in thiopurines drugs activation, was analyzed and resulted comparable between BJ-NSCs and AGS-NSCs (Figure 3D).

#### 3.2.2 JAK inhibitors

**FIGURE 4:**
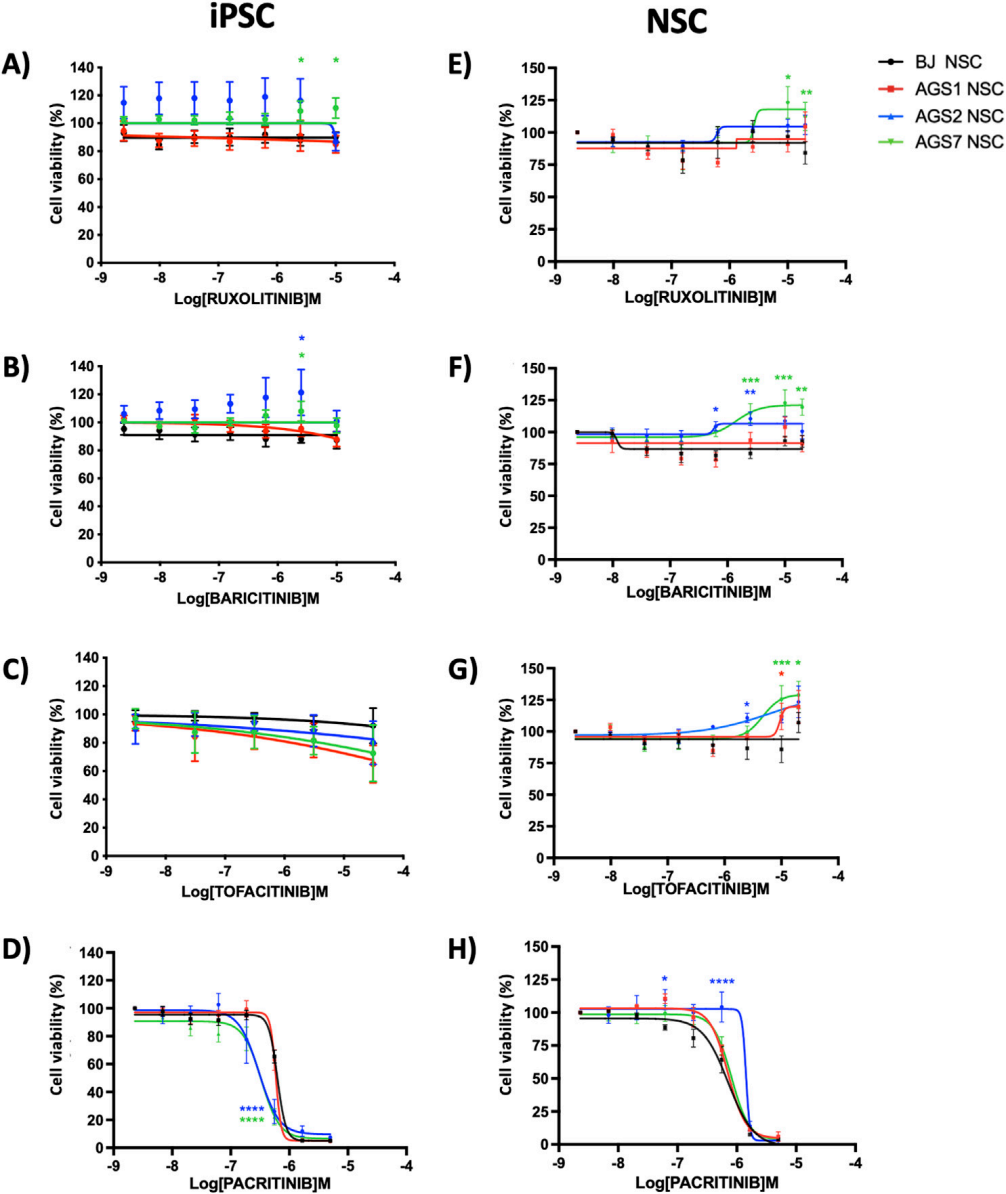
Dose-response curve (by MTT assay) after 72 h exposure of ruxolitinib, baricitinib, tofacitinib, pacritinib in iPSCs **(A–D)** and NSCs **(E–H)**. Seeding density: AGS2-iPSCs: 3.0 × 10^4^ cells/well, all other iPSCs and NSCs: 1.0 × 10^4^ cells/well in 96-well plate. The data are reported as percentage of cell viability as means ± standard error (SE) of 3 independent experiments performed in triplicate. Statistical analysis vs control BJ stem cells: *P < 0.05, **P < 0.01, ***P < 0.001, ****P < 0.0001, Two-way ANOVA and Bonferroni’s post-test. Green asterisks referred to AGS7- versus BJ-stem cells; blue asterisks to AGS2- versus BJ-stem cells; red asterisks to AGS1- versus BJ-stem cells.

Among JAKi, baricitinib, like ruxolitinib, is a non-selective JAK1/JAK2 inhibitor and is thus interfering with the IFNAR activation, acting through the JAK1 inhibition. Additional JAKi were included in the study to investigate the safety of these drugs associated with different JAKs selectivity profiles. In particular, tofacitinib is a JAK1/JAK3 inhibitor, whereas pacritinib acts as JAK2/FLTR3 inhibitor. With the exception of pacritinib, JAKi did not compromise iPSCs and NSCs viability (Figure 4). In iPSCs, a 3-day exposure to high concentration of ruxolitinib (>2.5 µM) increased cell viability in AGS7-iPSCs compared to control BJ-iPSC (Figure 4A, green asterisks). A similar increase in cell viability was observed for AGS7-iPSCs and AGS2-iPSCs when exposed to baricitinib at concentration of 2.5 µM (Figure 4B, green and blue asterisk, respectively). Exposure of NSCs to high concentrations of ruxolitinib (>10 μM), baricitinib (>2.5 μM) and tofacitinib (>10 μM) increased cell viability in AGS7-NSC compared to BJ-NSCs (Figures 4E–G, green asterisks); a similar result was observed for AGS2-NSCs versus BJ-NSCs treated with baricitinib at 0.6 μM and 2.5 μM (Figure 4F, blue asterisks) or tofacitinib at 2.5 μM (Figure 4G, blue asterisk) and also for AGS1-NSCs treated with tofacitinib at 10 μM (Figure 4G, red asterisk). Pacritinib was cytotoxic to all iPSC (IC_50_ ± SE, BJ-iPSC: 0.61 ± 0.03 µM; AGS1-iPSC: 0.58 ± 0.04 µM; AGS2-iPSC: 0.29 ± 0.13 µM; AGS7-iPSC: 0.35 ± 0.07 µM, Figure 4D) and all NSCs (IC_50_ ± SE: BJ-NSC: 0.63 ± 0.13 µM; AGS1-NSC: 0.75 ± 0.05 µM; AGS2-NSC: 1.05 ± 0.34 µM; AGS7-NSC: 0.81 ± 0.08 µM, Figure 4H).

Gene expression of *JAK1/STAT2* and *TYK2/STAT1*, crucial molecular targets involved in the JAKi pharmacodynamics and activated by the IFN receptors, were evaluated in iPSCs and NSCs. *JAK1, STAT1, TYK2* and *STAT2* showed comparable mRNA expression among iPSCs and among NSCs as well as between iPSCs and related NSCs with the exception of STAT2 that resulted higher in AGS7-iPSCs compared to AGS7-NSCs (Supplementary Figure S1).

#### 3.2.3 Antiretrovirals

**FIGURE 5:**
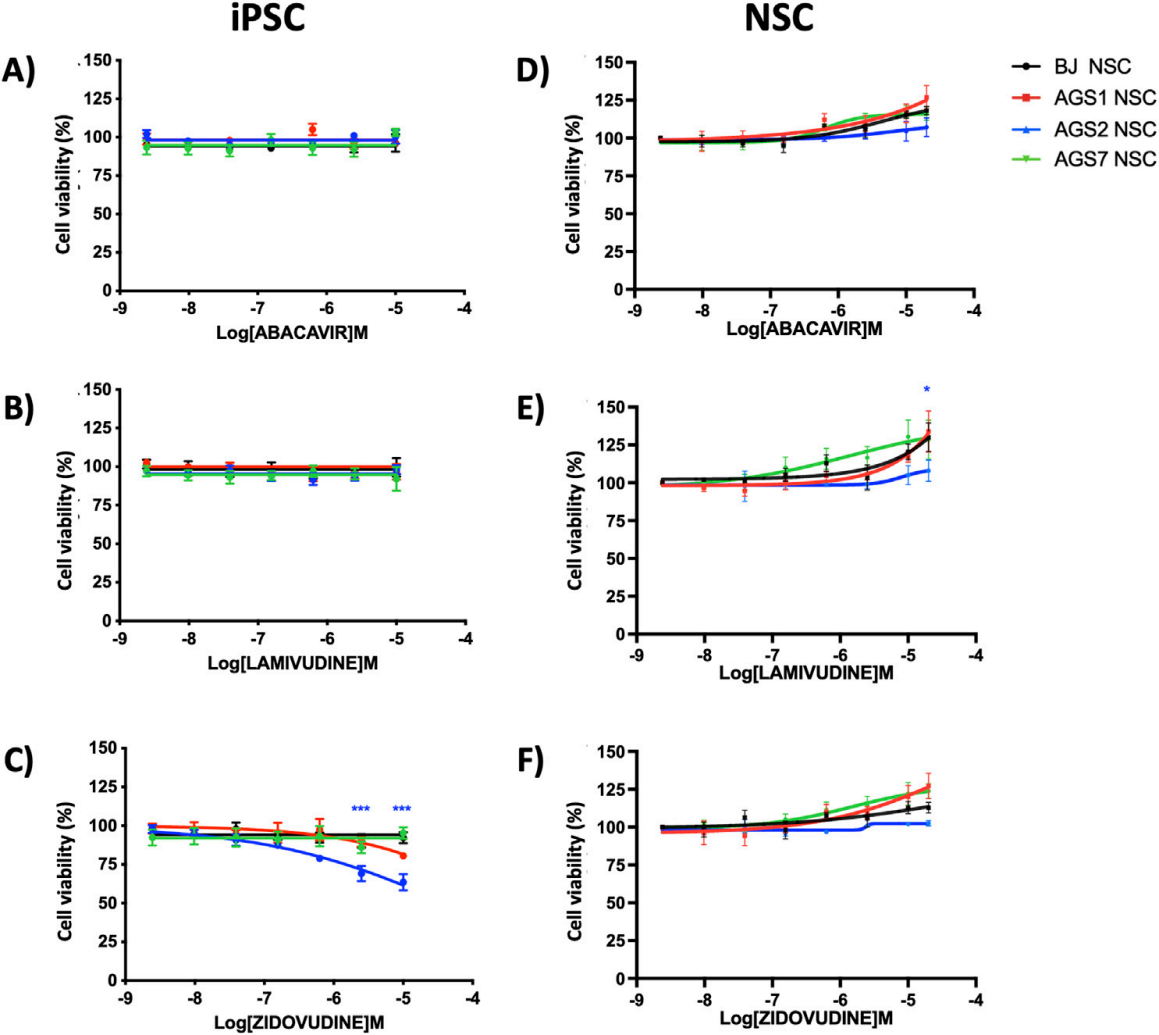
Dose-response curve (by MTT assay) after 72 h exposure with abacavir, lamivudine, zidovudine, in iPSCs **(A–C)** and NSCs **(D–F)**, respectively. The data are reported as percentage of cell viability as means ± standard error (SE) of 3 independent experiments performed in triplicate. Statistical analysis vs control BJ: *P < 0.05, ***P < 0.001, Two-way ANOVA and Bonferroni’s post-test. Blue asterisks reffered to AGS2- versus BJ-stem cells.

Abacavir sulfate and lamivudine did not show cytotoxicity on iPSCs (Figures 5A, B); survival of AGS2-iPSCs seemed to be affected by zidovudine at higher concentrations, although IC_50_ could not be calculated in the range of concentrations tested (Figure 5C); however, this drug effect was lost in AGS2-NSCs (Figure 5F). A trend toward a dose-dependent increase of cell viability after RTIs exposure was observed in NSCs. In the case of lamivudine, the viability of AGS2-NSCs was significantly lower than BJ-NSCs at the higher concentration tested (20 μM, Figure 5E). The expression of key genes involved in RTI drug pathways (*ADK* for abacavir, *DCK* for lamivudine, *TK1* for zidovudine) was comparable between iPSCs and derived NSCs for each cell line with the exception of *ADK*, whose expression level increased in AGS1-NSCs compared to AGS1-iPSCs (Supplementary Figure S1); however, no differences were found in terms of sensitivity to abacavir treatments.

## 4 Discussion

As a rare disease, cure of AGS is challenging for clinicians for many reasons, including the difficulty in conducting clinical trials with an adequate number of patients. Judging the efficacy of the therapeutic interventions across published studies and case-reports is difficult because of the different regimens attempted, the heterogeneity of the AGS subtypes and heterogeneity in the stage of the disease process at which treatment was started (Crow et al., 2020). A further complication in AGS study is represented by the lack of adequate animal models: AGS gene specific knockout mice models are useful but have the limitation that they cannot recapitulate all phenotypic features of diseases (Gray et al., 2015). In particular, they failed to recapitulate the strong central nervous system (CNS) involvement seen in AGS (Morita et al., 2004; Reijns et al., 2012; Bartsch et al., 2018). Other mouse models that mirror AGS neuropathology do not present the AGS causative genetic background (Akwa et al., 1998; Campbell et al., 1999; Viengkhou et al., 2024b). The importance of creating AGS patient-derived disease models to better understand inherited syndromes and to find ground-breaking therapies is becoming increasingly important in clinical and basic research. Patients’ neurons are not accessible, thus iPSCs and derived-NSCs offer an unprecedented opportunity to provide optimal patients cells surrogates, keeping the genetic background of the individuals. The iPSCs and NSCs represent a promising model for pharmacological studies, thanks to their ability to recapitulate the tissue characteristics maintaining the patient’s genetic features. In AGS patients brain damage occurs mainly in the early phases of neuronal development and symptoms occur months to years after birth when cells are differentiated (Orcesi et al., 2009); it is crucial to study the safety profile of any drug proposed for AGS, even in stem cells, since new functional neurons can be generated from NSCs pool residing in neural niche (Göritz and Frisén, 2012). Indeed, the use of iPSCs allowed to conduct safety studies on the AGS-iPSCs themselves, or on derived multipotent cellular model such as NSCs (Genova et al., 2019). In the current study we tested the cytotoxicity of iPSCs and NSCs after the exposure to “broad active” immunosuppressive drugs used in the clinical practice, such as thiopurines and glucocorticoids, able to manage and reduce the inflammatory condition (Crow et al., 2014; Crow et al., 2020) and new molecules proposed as promising AGS therapies, such as RTI and JAKi, which demonstrated to be beneficial for AGS patients (Tonduti et al., 2020; Vanderver et al., 2020). In this study, we used AGS patient-derived iPSC cell lines generated and fully characterized by Ferraro and collogues (Ferraro et al., 2019a; Ferraro et al., 2019b; Masneri et al., 2019) and differentiated them to NSCs. The expression of pluripotency gene markers for different grade of stemness was assessed in both iPSCs and NSCs. The decreased *OCT4* gene expression, due to the gradual methylation of its promoter and proximal enhancer region during the differentiation processes (Lee et al., 2010), combined to the increased levels of *SOX1* and *PAX6 (*transcription factors involved in the early steps of neurogenesis (Yan et al., 2013)) confirmed the proper differentiation of iPSC into NSCs. *SOX2* and *Nestin* levels of expression were found to be comparable between iPSCs and NSCs, as expected by literature (Ellis et al., 2004). We compared the metabolic activity of stem cells. IPSC showed a reduced entry into proliferation due to a higher G0 percentage. Moreover, among iPSCs, AGS2- showed a lower percentage of cells in G2 phase compared to healthy control BJ-iPSC. Metabolic activity of iPSCs under basal condition, measured by MTT assay, was previously reported (Genova et al., 2020), showing a reduced survival in AGS2-iPSCs compared to other iPSCs. AGS2 patients are characterized by a reduction in *RNASEH2B* transcript and related protein levels (Garau et al., 2021). The lower proliferation of *RNASEH2B* deficient embryonic mouse cells was also reported by Hiller and collaborators (Hiller et al., 2012). RNASE H2 is an endonuclease that degrades RNA within an RNA:DNA hybrids (Cerritelli and Crouch, 2009) and is specifically responsible for removing single ribonucleotides that are misincorporated into DNA during DNA replication (Reijns et al., 2012). *RNASEH2B* mutation leads to a reduction of RNASE H2 activity and, consequently, to aberrant accumulation of ribonucleotides into genomic DNA (Rabe, 2013). It was demonstrated that RNASE H2 is able to exert its functions specifically in the G2 phase of the cell cycle (Lockhart et al., 2019). Among NSCs, the *RNASEH2B* deficient (hypomorphic) line did not show a different metabolic activity compared to other NSCs suggesting that the defective proliferative process observed in iPSCs could be lost at a higher degree of neural differentiation for unknown reasons.

AGS are inherited type I interferonopathies and affect the nervous tissue linking a degenerative pattern to an inflammatory phenotype; for this reason, initial empirical therapeutic approaches involved drugs targeting the immune system, such as glucocorticoids and thiopurines. The primary mechanism of action of dexamethasone relies on by binding to the ubiquitously expressed glucocorticoid receptor, encoded by the *NR3C1* gene. After glucocorticoid binding, the receptor complex migrates to the nucleus, binding to the DNA at the *glucocorticoid response elements* thus regulating expression of specific genes (Tischner and Reichardt, 2007; Reichardt et al., 2021). Previous results on AGS- and BJ-iPSCs and dexamethasone treatment showed lack of cytotoxic activity (Genova et al., 2020); here we confirmed the loss glucocorticoid cytotoxicity of NSCs due to the lack of *NR3C1* expression, giving a rational for the safe use of glucocorticoids on stem cells.

Previous results on iPSCs have shown thiopurines induced cytotoxicity in both BJ- and AGS-iPSCs (Genova et al., 2020). In this study, AGS2- and AGS7-NSCs were sensitive to thioguanine, whereas the AGS1-NSC cells presented a low cytotoxicity after thioguanine exposure, showing the 70% of viability after 72 h of treatment. In contrast, no cytotoxicity was detected after mercaptopurine treatment likely because of the different cellular metabolism of these molecules. Mercaptopurine seemed safer than thioguanine for NSCs. Since it has a far more complex intracellular metabolism and the reaction mediated by *HPRT1* is the first of the multiple steps in the activation to thioguanine-nucleotides (Franca et al., 2021), it is possible that the cytotoxic effect of mercaptopurine could not be observed after 72 h incubation. The distinct sensitivity profiles of BJ- and AGS-NSCs suggest that pathogenic mutations present in AGS patients may influence thioguanine cytotoxicity. In 1999, the group of Prof. Evans (St. Jude Children’s Research Hospital, Memphis, United States) showed that incorporation of thioguanine nucleotides into the DNA strand significantly inhibited (80%–90%) human RNase H-catalyzed RNA cleavage from DNA-RNA heteroduplexes in leukemic cell lines, indicating that thiopurine mechanism of cytotoxicity may involve interference with this component of the replication machinery (Krynetskaia et al., 1999). This inhibition resembles the condition of AGS2 patients; however, it is not clear how the lack RNASEH2B activity in AGS2 patients could enhance thioguanine toxicity. *HPRT1* mRNA was similarly expressed in BJ–and AGS-NSCs; therefore, it would be important to further assess either the HPRT1 protein expression or the metabolite levels in NSCs for a deeper characterization of thioguanine metabolism in these cell lines. Of note, an overall shift towards resistance to thiopurines was observed in NSCs compared to iPSCs in AGS-patient-derived cells (Genova et al., 2020). One possible explanation could be the unique cell cycle characteristics of iPSC. iPSCs have a rapid division time (16–18 h) and a very short G1 phase (∼2.5 h). As development proceeds, the cell cycle length of neural progenitor cells increases (to up to ∼18 h), due to a four-fold increase in the length of G1 (Liu et al., 2019). The rapid cell cycle of iPSCs might make them more sensitive to cytotoxic agents such as thiopurines, which target the S phase of the cell cycle. When thiopurines are incorporated into the DNA during the S phase, they can cause cell cycle arrest. Additionally, drug resistance mechanisms such as enhanced efflux through activation of transmembrane proteins, increased detoxification and activation of anti-apoptosis or cell cycle arrest can not be ruled out. These mechanisms could contribute to the varying sensitivity.

With advanced understanding of molecular pathogenic processes in AGS, pharmacological strategies have shifted towards more targeted approaches, including repurposing currently approved drugs in use for other diseases (i.e., JAKi and RTIs) (Crow et al., 2020; Genova et al., 2020). The anti-proliferative and immunosuppressive effects of JAKi on immune cells are expected (Tonduti et al., 2020), whereas the efficacy and safety profiles of JAKi on patients NSCs and functional neurons need to be better investigated. The JAK/STAT signaling pathway plays a key role in the balance between NSCs quiescence and proliferation (Garza et al., 2008; Kim et al., 2010; Nicolas et al., 2013), in neurogenesis versus gliogenesis lineage decisions (Bonni et al., 1997; Nakanishi et al., 2007), in differentiation of NSCs into mature neurons (Kim et al., 2010) and in synaptic plasticity (Nicolas et al., 2012; Nicolas et al., 2013; Yasuda et al., 2021). In AGS patients, it is thus necessary to assess whether exposure to JAKi could be cytotoxic for NSCs. The significant cytotoxicity observed on iPSCs and NSCs suggest an unsafe use only of pacritinib for AGS. With IC_50_ in the low micromolar range, pacritinib has been shown to affect the viability of brain tumor-initiating cells and sphere forming capacity in preclinical studies (Jensen et al., 2017). The different cytotoxic profile of pacritinib can be attributed to its affinity for different targets such as IRAK1 and FLT3. In particular, FLT3 is a tyrosine kinase receptor expressed on the surface of multipotent stem cells (e.g.,: hematopoietic stem cells), playing an important role in the survival and proliferation of these cells (Verstovsek and Komrokji, 2015). The exposure of iPSCs and NSCs to other JAKi was not cytotoxic. In contrast, an unexpected increase in cell viability was observed in AGS patient-derived NSCs compared to the control BJ stem cells at high concentrations of JAKi (ruxolitinib, baricitinib, tofacitinib). The biological meaning of this finding is unclear. First, it would be important to discriminate whether JAKi promote the stem cell proliferation or the cell metabolism. This can be achieved by using more direct measurements of cell growth dynamics, such as [3H]-thymidine or BrdU incorporation assays, in both JAKi-treated and untreated cells to evaluate the impact on cell division. Additionally, studying autophagic and apoptotic processes, as well as ROS production following JAKi exposure, will provide further mechanistic insights. A strong relationship between autoimmune diseases, neuroinflammation and mitochondrial dysfunctions has been reported by several authors (Fang et al., 2016; Gambardella et al., 2019; van Horssen et al., 2019; Barrera et al., 2021; Dragoni et al., 2022). In rheumatoid arthritis, tofacitinib significantly increased oxidative phosphorylation, ATP production, and the maximal respiratory capacity and the respiratory reserve in primary synovial fibroblasts, suggesting JAK/STAT signaling as a mediator of the complex interplay between inflammation and cellular metabolism (McGarry et al., 2018). It could therefore be hypothesized that in this complex interplay the use of JAKi can reduce cells oxidative stress thus increasing metabolic response. Second, the unexpected increase in cell viability in patient-derived cells was observed at µM concentrations of JAKi. In healthy adult subjects receiving ruxolitinib 50 mg once daily or baricitinib 5 mg two times a day for 10 days, plasma concentrations are ∼3 μM and 146 nM respectively (Shi et al., 2011; Shi et al., 2014). In a pediatric AGS patient, ruxolitinib was started at 2.5 mg twice daily and increased to 5 mg BID 10 weeks later (Cattalini et al., 2021), likely bringing to concentrations in the µM range. Although JAKi are considered highly selective for some members of the JAK family, as the intracellular concentration of these drugs increases, a loss of selectivity occurs due to the interaction with ATP binding sites of other kinases (Lin et al., 2020). Conducting single-cell RNA sequencing (scRNAseq) on JAKi-treated and untreated iPSCs and NSCs could help identify the affected pathways and show how these pathways might change across different differentiation states. Third, it is possible that in the genetic settings of AGS, the higher concentrations of JAKi altered the equilibrate cross-talk between the JAK/STAT signaling and other proliferative pathway, potentially in favor of the latter. STAT proteins are crucial in regulating cell cycle progression, growth arrest and apoptosis (Bromberg and Darnell, 2000). Through the receptor-mediated activation of STAT1, IFNs inhibit cell proliferation (Bromberg et al., 1996; Liu et al., 2023). Consequently, high concentrations of JAKi, might interfere with the transcription of antiproliferative genes. A comprehensive protein profiling of the IFN signaling pathway, in particular comparing the levels of IFNs in cell surnatants as well as the expression of IFNARs and STATs between AGS- and BJ- stem cell derived cells, would be useful for an accurate interpretation of these results. Moreover, it would be also interesting to examine the ISGs profile of the stem cells. Analysis of genes expression of the JAK/STAT pathway show only the difference in *STAT2* gene expression between iPSCs and NSCs in AGS7 patient. This observation is in line with the JAK-STAT pathway being present in all cell types and mediating responses to a wide array of cytokines, suggesting a fundamental role in cellular communication and homeostasis across different tissues (Hu et al., 2023).

In AGS patient-derived iPSCs lines, RTIs treatment did not affect AGS1-, AGS7- and BJ-iPSCs survival. The AGS2-iPSCs viability was significantly decreased after 3-day exposure at higher zidovudine concentrations, in contrast to other iPSCs; this difference was no longer observable between AGS2-NSCs and the other NSCs. However, expression of TK1, a key target gene involved in zidovudine activation, was comparable among iPSCs and NSCs. The increased cytotoxic effect of zidovudine on AGS2 patient-derived iPSCs suggests a disease-specific genetic contribution to drug sensitivity (Crow et al., 2014). In conclusion, the JAKi and RTIs drugs tested did not affect iPSCs and NSCs survival.

This study presents some limitations. The small number of AGS patients and controls included in the study, due to the low incidence rate of AGS disease (Aicardi and Goutières, 1984; Lebon et al., 1988) as well as the complexity and labor-intensive nature of cell line generation, may restrict the data robustness of the findings. Also the use of iPSCs/NSCs as a cellular model to study the effects of immunomodulatory/antiretroviral agents, which are effective on immune cells (Aquaro et al., 2020; Strzelec et al., 2023) has to be taken carefully. It is important to acknowledge that the cytotoxic results obtained in iPSCs and NSCs required additional mechanistic studies and functional assays to be correctly interpret; they may not be representative of the cytotoxic profiles of differentiated cells, due to various reasons, such as proliferation rate, cell cycle, epigenetics, relative density of drug target and enzyme expression profile (Liang and Zhang, 2013). Of great importance will be the differentiation of NSCs to tissues involved in the pathogenesis of AGS, such as the immune or nervous system cells (Barth et al., 1999; Kavanagh et al., 2016; Thomas et al., 2017; Viengkhou et al., 2024a). In a future perspective, it will be interesting to evaluate *in vitro* the impact of JAKi/RTI on neurons and microglia, particularly on astrocytes proposed as key players in AGS pathology since they are thought to be the primary cells that produce IFN-α in CNS. Cuadrado et al. demonstrated that when the AGS gene *TREX1* was silenced in astrocytes, a significant induction of IFN-α expression, ISG signaling and release of pro-inflammatory cytokines and chemokines was observed (Van Heteren et al., 2008; Cuadrado et al., 2015). Understanding the contribution of cell-autonomous and non-cell-autonomous role of astrocytes will help reveal mechanisms underlying interferonopathy in AGS (Sase et al., 2018). Co-culture experiments could involve treating patient-derived astrocytes with JAKi and then using the culture supernatant as a conditioned medium for neurons to observe if their conditions improve. Nowadays, there are still several limitations on working with iPSCs-derived neurons, including time, costs, technical issues such as the variability in the neuronal pool for high throughput screening and reproducibility of results among iPSCs clones of the same individual (Dolmetsch and Geschwind, 2011; Yoshihara et al., 2017). However, iPSCs and iPSC derived cells carry the AGS patient’s specific genomic background and may be informative of drug effects in this pathogenic context.

## 5 Conclusion

In conclusion, this work highlights the importance in basic and clinical research of creating patient-specific disease models to better understand inherited syndromes and to deeper our knowledge on innovative therapeutic approaches. We used patient-specific iPSCs as model for AGS, obtained by reprogramming patients’ primary cells and differentiated into NSCs. AGS patient-derived *in vitro* model established has proven to be suitable for studying the safety profile of a panel of drugs on NSCs, being a cell type otherwise inaccessible. According to our results, all drug tested, in particular glucocorticoids, JAKi and RTI but not thioguanine and pacritinib, could be safe for NSCs of AGS patients.

As future prospective, it may be interesting to broaden the panel of drug tested on NSCs-derived neuronal cells. Additionally, considering the involvement of the immune component in AGS, it may be beneficial to analyze the effects of these novel drugs in neuronal co-cultures derived from NSCs, alongside cells from the immune system.

## Data Availability

The data supporting the findings of this study are available from the corresponding author upon reasonable request.

## References

[B1] Aicardi J. Goutières F. (1984). A Progressive familial encephalopathy in infancy with calcifications of the basal ganglia and chronic cerebrospinal fluid lymphocytosis. Ann. Neurology 15 (1), 49–54. 10.1002/ana.410150109 6712192

[B2] Akwa Y. Hassett D. E. Eloranta M. L. Sandberg K. Masliah E. Powell H. (1998). Transgenic expression of IFN-α in the central nervous system of mice Protects against Lethal Neurotropic viral Infection but Induces inflammation and Neurodegeneration. J. Immunol. 161 (9), 5016–5026. 10.4049/jimmunol.161.9.5016 9794439

[B3] Ali E. Ferraro R. M. Lanzi G. Masneri S. Piovani G. Mazzoldi E. L. (2020). Generation of induced pluripotent stem cell (iPSC) lines from a Joubert syndrome patient with compound heterozygous mutations in C5orf42 gene. Stem Cell Res. 49, 102007. 10.1016/j.scr.2020.102007 33010677

[B4] Al Mutairi F. Alfadhel M. Nashabat M. El-Hattab A. W. Ben-Omran T. Hertecant J. (2018). Phenotypic and molecular Spectrum of Aicardi-Goutières syndrome: a study of 24 patients. Pediatr. Neurol. 78, 35–40. 10.1016/j.pediatrneurol.2017.09.002 29239743

[B5] Aquaro S. Borrajo A. Pellegrino M. Svicher V. (2020). Mechanisms underlying of antiretroviral drugs in different cellular reservoirs with a focus on macrophages. Virulence 11 (1), 400–413. 10.1080/21505594.2020.1760443 32375558 PMC7219522

[B6] Aso H. Ito J. Koyanagi Y. Sato K. (2019). Comparative Description of the expression profile of interferon-stimulated genes in multiple cell lineages targeted by HIV-1 Infection. Front. Microbiol. 10, 429. 10.3389/fmicb.2019.00429 30915053 PMC6423081

[B7] Barrera M.-J. Aguilera S. Castro I. Carvajal P. Jara D. Molina C. (2021). Dysfunctional mitochondria as critical players in the inflammation of autoimmune diseases: potential role in Sjögren’s syndrome. Autoimmun. Rev. 20 (8), 102867. 10.1016/j.autrev.2021.102867 34118452

[B8] Barth P. G. Walter A. van Gelderen I. (1999). Aicardi-Goutières syndrome: a genetic microangiopathy? Acta Neuropathol. 98 (2), 212–216. 10.1007/s004010051071 10442562

[B9] Bartsch K. Damme M. Regen T. Becker L. Garrett L. Hölter S. M. (2018). RNase H2 loss in Murine astrocytes results in cellular Defects Reminiscent of nucleic acid-mediated Autoinflammation. Front. Immunol. 9, 587. 10.3389/fimmu.2018.00587 29662492 PMC5890188

[B10] Bonni A. Sun Y. Nadal-Vicens M. Bhatt A. Frank D. A. Rozovsky I. (1997). Regulation of gliogenesis in the Central nervous system by the JAK-STAT signaling pathway. Science 278 (5337), 477–483. 10.1126/science.278.5337.477 9334309

[B11] Bromberg J. Darnell J. E. (2000). The role of STATs in transcriptional control and their impact on cellular function. Oncogene 19 (21), 2468–2473. 10.1038/sj.onc.1203476 10851045

[B12] Bromberg J. F. Horvath C. M. Wen Z. Schreiber R. D. Darnell J. E. (1996). Transcriptionally active Stat1 is required for the antiproliferative effects of both interferon alpha and interferon gamma. Proc. Natl. Acad. Sci. U. S. A. 93 (15), 7673–7678. 10.1073/pnas.93.15.7673 8755534 PMC38805

[B13] Campbell I. L. Krucker T. Steffensen S. Akwa Y. Powell H. C. Lane T. (1999). Structural and functional neuropathology in transgenic mice with CNS expression of IFN-alpha. Brain Res. 835 (1), 46–61. 10.1016/s0006-8993(99)01328-1 10448195

[B14] Cattalini M. Galli J. Zunica F. Ferraro R. M. Carpanelli M. Orcesi S. (2021). Case report: the JAK-inhibitor ruxolitinib Use in Aicardi-Goutieres syndrome due to *ADAR1* mutation. Front. Pediatr. 9, 725868. 10.3389/fped.2021.725868 34778129 PMC8578119

[B15] Cerritelli S. M. Crouch R. J. (2009). Ribonuclease H: the enzymes in eukaryotes. FEBS J. 276 (6), 1494–1505. 10.1111/j.1742-4658.2009.06908.x 19228196 PMC2746905

[B16] Crow M. K. (2003). Type I interferon and autoimmune disease. Autoimmunity 36 (8), 445–446. 10.1080/08916930310001625961 14984020

[B17] Crow Y. J. Chase D. S. Lowenstein Schmidt J. Szynkiewicz M. Forte G. M. A. Gornall H. L. (2015). Characterization of human disease phenotypes associated with mutations in TREX1, RNASEH2A, RNASEH2B, RNASEH2C, SAMHD1, ADAR, and IFIH1. Am. J. Med. Genet. Part A 167 (2), 296–312. 10.1002/ajmg.a.36887 PMC4382202 25604658

[B18] Crow Y. J. Shetty J. Livingston J. H. (2020). Treatments in Aicardi–Goutières syndrome. Dev. Med. & Child Neurology 62 (1), 42–47. 10.1111/dmcn.14268 31175662

[B19] Crow Y. J. Vanderver A. Orcesi S. Kuijpers T. W. Rice G. I. (2014). Therapies in Aicardi–Goutières syndrome. Clin. Exp. Immunol. 175 (1), 1–8. 10.1111/cei.12115 23607857 PMC3898548

[B93] Cuadrado E. Michailidou I. van Bodegraven E. J. Jansen M. H. Sluijs J. A. Geerts D. (2015). Phenotypic variation in Aicardi–Goutières syndrome explained by cell‐specific IFN‐stimulated gene response and cytokine release. J. Immunol. 194, 3623–3633. 10.4049/jimmunol.1401334 25769924

[B20] Dolmetsch R. Geschwind D. H. (2011). The human brain in a dish: the promise of iPSC-derived neurons. Cell 145 (6), 831–834. 10.1016/j.cell.2011.05.034 21663789 PMC3691069

[B21] Dragoni F. Garau J. Sproviero D. Orcesi S. Varesio C. De Siervi S. (2022). Characterization of mitochondrial alterations in Aicardi–Goutières patients mutated in RNASEH2A and RNASEH2B genes. Int. J. Mol. Sci. 23, 14482. 10.3390/ijms232214482 36430958 PMC9692803

[B22] Ellis P. Fagan B. M. Magness S. T. Hutton S. Taranova O. Hayashi S. (2004). SOX2, a persistent marker for Multipotential neural stem cells derived from embryonic stem cells, the Embryo or the adult. Dev. Neurosci. 26 (2-4), 148–165. 10.1159/000082134 15711057

[B23] Fang C. Wei X. Wei Y. (2016). Mitochondrial DNA in the regulation of innate immune responses. Protein & Cell 7 (1), 11–16. 10.1007/s13238-015-0222-9 26498951 PMC4707157

[B24] Ferraro R. M. Lanzi G. Masneri S. Barisani C. Piovani G. Savio G. (2019a). Generation of three iPSC lines from fibroblasts of a patient with Aicardi Goutières Syndrome mutated in TREX1. Stem Cell Res. 41, 101580. 10.1016/j.scr.2019.101580 31644995

[B25] Ferraro R. M. Masneri S. Lanzi G. Barisani C. Piovani G. Savio G. (2019b). Establishment of three iPSC lines from fibroblasts of a patient with Aicardi Goutières syndrome mutated in RNaseH2B. Stem Cell Res. 41, 101620. 10.1016/j.scr.2019.101620 31678772

[B26] Franca R. Braidotti S. Stocco G. Decorti G. (2021). Understanding thiopurine methyltransferase polymorphisms for the targeted treatment of hematologic malignancies. Expert Opin. Drug Metabolism & Toxicol. 17 (10), 1187–1198. 10.1080/17425255.2021.1974398 34452592

[B27] Fryer A. L. Abdullah A. Taylor J. M. Crack P. J. (2021). The complexity of the cGAS-STING pathway in CNS Pathologies. Front. Neurosci. 15, 621501. 10.3389/fnins.2021.621501 33633536 PMC7900568

[B28] Galli J. Cattalini M. Loi E. Ferraro R. M. Giliani S. Orcesi S. (2023). Treatment response to Janus kinase inhibitor in a child affected by Aicardi-Goutières syndrome. Clin. Case Rep. 11 (8), e7724. 10.1002/ccr3.7724 37534202 PMC10390657

[B29] Galli J. Gavazzi F. De Simone M. Giliani S. Garau J. Valente M. (2018). Sine causa tetraparesis: a pilot study on its possible relationship with interferon signature analysis and Aicardi Goutières syndrome related genes analysis. Med. Baltim. 97 (52), e13893. 10.1097/MD.0000000000013893 PMC6314769 30593198

[B30] Gambardella S. Limanaqi F. Ferese R. Biagioni F. Campopiano R. Centonze D. (2019). ccf-mtDNA as a potential Link between the brain and immune system in Neuro-Immunological disorders. Front. Immunol. 10, 1064. 10.3389/fimmu.2019.01064 31143191 PMC6520662

[B31] Garau J. Masnada S. Dragoni F. Sproviero D. Fogolari F. Gagliardi S. (2021). Case report: novel compound heterozygous RNASEH2B mutations cause Aicardi–Goutières syndrome. Front. Immunol. 12, 672952. 10.3389/fimmu.2021.672952 33981319 PMC8107470

[B32] Garza J. C. Guo M. Zhang W. Lu X.-Y. (2008). Leptin increases adult Hippocampal neurogenesis *in vivo* and *in vitro* . J. Biol. Chem. 283 (26), 18238–18247. 10.1074/jbc.M800053200 18367451 PMC2440628

[B33] Genova E. Cavion F. Lucafò M. Leo L. D. Pelin M. Stocco G. (2019). Induced pluripotent stem cells for therapy personalization in pediatric patients: focus on drug-induced adverse events. World J. stem cells 11 (12), 1020–1044. 10.4252/wjsc.v11.i12.1020 31875867 PMC6904863

[B34] Genova E. Cavion F. Lucafò M. Pelin M. Lanzi G. Masneri S. (2020). Biomarkers and Precision therapy for primary Immunodeficiencies: an *in vitro* study based on induced pluripotent stem cells from patients. Clin. Pharmacol. & Ther. 108 (2), 358–367. 10.1002/cpt.1837 32243572

[B35] Göritz C. Frisén J. (2012). Neural stem cells and neurogenesis in the adult. Cell Stem Cell 10 (6), 657–659. 10.1016/j.stem.2012.04.005 22704503

[B36] Gray E. E. Treuting P. M. Woodward J. J. Stetson D. B. (2015). Cutting Edge: cGAS is required for Lethal autoimmune disease in the Trex1-deficient mouse model of Aicardi–Goutières syndrome. J. Immunol. 195 (5), 1939–1943. 10.4049/jimmunol.1500969 26223655 PMC4546858

[B37] Hiller B. Achleitner M. Glage S. Naumann R. Behrendt R. Roers A. (2012). Mammalian RNase H2 removes ribonucleotides from DNA to maintain genome integrity. J. Exp. Med. 209 (8), 1419–1426. 10.1084/jem.20120876 22802351 PMC3409502

[B38] Hu Q. Bian Q. Rong D. Wang L. Song J. Huang H. S. (2023). JAK/STAT pathway: Extracellular signals, diseases, immunity, and therapeutic regimens. Front. Bioeng. Biotechnol. 11, 1110765. 10.3389/fbioe.2023.1110765 36911202 PMC9995824

[B39] Iro M. A. Martin N. G. Absoud M. Pollard A. J. (2017). Intravenous immunoglobulin for the treatment of childhood encephalitis. Cochrane database Syst. Rev. 10 (10), CD011367. 10.1002/14651858.CD011367.pub2 28967695 PMC6485509

[B40] Jensen K. V. Cseh O. Aman A. Weiss S. Luchman H. A. (2017). The JAK2/STAT3 inhibitor pacritinib effectively inhibits patient-derived GBM brain tumor initiating cells *in vitro* and when used in combination with temozolomide increases survival in an orthotopic xenograft model. PLoS One 12 (12), e0189670. 10.1371/journal.pone.0189670 29253028 PMC5734728

[B41] Kavanagh D. McGlasson S. Jury A. Williams J. Scolding N. Bellamy C. (2016). Type I interferon causes thrombotic microangiopathy by a dose-dependent toxic effect on the microvasculature. Blood 128 (24), 2824–2833. 10.1182/blood-2016-05-715987 27663672 PMC5159705

[B42] Kim Y. H. Chung J.-I. Woo H. G. Jung Y.-S. Lee S. H. Moon C.-H. (2010). Differential regulation of proliferation and differentiation in neural Precursor cells by the Jak pathway. Stem Cells 28 (10), 1816–1828. 10.1002/stem.511 20979137

[B43] Krynetskaia N. F. Krynetski E. Y. Evans W. E. (1999). Human RNase H-mediated RNA cleavage from DNA-RNA Duplexes is inhibited by 6-Deoxythioguanosine incorporation into DNA. Mol. Pharmacol. 56 (4), 841–848. 10.1016/s0026-895x(24)12548-5 10496969

[B44] Lama L. Adura C. Xie W. Tomita D. Kamei T. Kuryavyi V. (2019). Development of human cGAS-specific small-molecule inhibitors for repression of dsDNA-triggered interferon expression. Nat. Commun. 10 (1), 2261. 10.1038/s41467-019-08620-4 31113940 PMC6529454

[B45] Landrieu P. Baets J. De Jonghe P. (2013). Hereditary motor-sensory, motor, and sensory neuropathies in childhood. Handb. Clin. Neurology 113, 1413–1432. 10.1016/B978-0-444-59565-2.00011-3 23622364

[B46] La Piana R. Uggetti C. Roncarolo F. Vanderver A. Olivieri I. Tonduti D. (2016). Neuroradiologic patterns and novel imaging findings in Aicardi-Goutières syndrome. Neurology 86 (1), 28–35. 10.1212/WNL.0000000000002228 26581299 PMC4731289

[B47] Lebon P. Badoual J. Ponsot G. Goutières F. Hémeury-Cukier F. Aicardi J. (1988). Intrathecal synthesis of interferon-alpha in infants with progressive familial encephalopathy. J. Neurological Sci. 84 (2), 201–208. 10.1016/0022-510x(88)90125-6 2837539

[B48] Lee S.-H. Jeyapalan J. N. Appleby V. Mohamed Noor D. A. Sottile V. Scotting P. J. (2010). Dynamic methylation and expression of Oct4 in early neural stem cells. J. Anat. 217 (3), 203–213. 10.1111/j.1469-7580.2010.01269.x 20646110 PMC2972534

[B49] Liang G. Zhang Y. (2013). Genetic and epigenetic variations in iPSCs: potential causes and implications for application. Cell stem Cell 13 (2), 149–159. 10.1016/j.stem.2013.07.001 23910082 PMC3760008

[B50] Lin C. M. Cooles F. A. Isaacs J. D. (2020). Basic mechanisms of JAK inhibition. Mediterr. J. Rheumatology 31 (Suppl. 1), 100–104. 10.31138/mjr.31.1.100 PMC7361186 32676567

[B51] Liu A. Ying S. (2023). Aicardi-Goutières syndrome: a monogenic type I interferonopathy. Scand. J. Immunol. 98 (4), e13314. 10.1111/sji.13314 37515439

[B52] Liu L. Michowski W. Kolodziejczyk A. Sicinski P. (2019). The cell cycle in stem cell proliferation, pluripotency and differentiation. Nat. Cell Biol. 21 (9), 1060–1067. 10.1038/s41556-019-0384-4 31481793 PMC7065707

[B53] Liu Z. Fan Z. Wang R. Li X. Chen H. Wang J. (2023). IFN-*α*-2b reduces Postoperative Arthrofibrosis in Rats by inhibiting fibroblast proliferation and migration through STAT1/p21 signaling pathway. Mediat. Inflamm. 2023, 1699946. 10.1155/2023/1699946 PMC10008118 36915717

[B54] Livingston J. H. Crow Y. J. (2016). Neurologic phenotypes associated with mutations in TREX1, RNASEH2A, RNASEH2B, RNASEH2C, SAMHD1, ADAR1, and IFIH1: Aicardi–Goutières syndrome and beyond. Neuropediatrics 47 (06), 355–360. 10.1055/s-0036-1592307 27643693

[B55] Lockhart A. Pires V. B. Bento F. Kellner V. Luke-Glaser S. Yakoub G. (2019). RNase H1 and H2 are Differentially regulated to process RNA-DNA hybrids. Cell Rep. 29 (9), 2890–2900. 10.1016/j.celrep.2019.10.108 31775053

[B56] Masneri S. Lanzi G. Ferraro R. M. Barisani C. Piovani G. Savio G. (2019). Generation of three isogenic induced Pluripotent Stem Cell lines (iPSCs) from fibroblasts of a patient with Aicardi Goutières Syndrome carrying a c.2471G>A dominant mutation in IFIH1 gene. Stem Cell Res. 41, 101623. 10.1016/j.scr.2019.101623 31698194

[B57] McGarry T. Orr C. Wade S. Biniecka M. Wade S. Gallagher L. (2018). JAK/STAT Blockade alters synovial Bioenergetics, mitochondrial function, and proinflammatory mediators in rheumatoid arthritis. Arthritis & Rheumatology 70 (12), 1959–1970. 10.1002/art.40569 29790294

[B58] Michalska A. Blaszczyk K. Wesoly J. Bluyssen H. A. R. (2018). A positive Feedback Amplifier Circuit that Regulates interferon (IFN)-Stimulated gene expression and controls type I and type II IFN responses. Front. Immunol. 9, 1135. 10.3389/fimmu.2018.01135 29892288 PMC5985295

[B59] Morita M. Stamp G. Robins P. Dulic A. Rosewell I. Hrivnak G. (2004). Gene-targeted mice lacking the Trex1 (DNase III) 3'-->5' DNA exonuclease develop inflammatory myocarditis. Mol. Cell. Biol. 24 (15), 6719–6727. 10.1128/MCB.24.15.6719-6727.2004 15254239 PMC444847

[B60] Nakanishi M. Niidome T. Matsuda S. Akaike A. Kihara T. Sugimoto H. (2007). Microglia-derived interleukin-6 and leukaemia inhibitory factor promote astrocytic differentiation of neural stem/progenitor cells. Eur. J. Neurosci. 25 (3), 649–658. 10.1111/j.1460-9568.2007.05309.x 17328769

[B61] Nicolas C. S. Amici M. Bortolotto Z. A. Doherty A. Csaba Z. Fafouri A. (2013). The role of JAK-STAT signaling within the CNS. JAK-STAT 2 (1), e22925. 10.4161/jkst.22925 24058789 PMC3670265

[B62] Nicolas C. S. Peineau S. Amici M. Csaba Z. Fafouri A. Javalet C. (2012). The Jak/STAT pathway is involved in synaptic plasticity. Neuron 73 (2), 374–390. 10.1016/j.neuron.2011.11.024 22284190 PMC3268861

[B63] Onomoto K. Onoguchi K. Yoneyama M. (2021). Regulation of RIG-I-like receptor-mediated signaling: interaction between host and viral factors. Cell Mol. Immunol. 18 (3), 539–555. 10.1038/s41423-020-00602-7 33462384 PMC7812568

[B64] Orcesi S. La Piana R. Fazzi E. (2009). Aicardi–goutières syndrome. Br. Med. Bull. 89 (1), 183–201. 10.1093/bmb/ldn049 19129251

[B65] Pulliero A. Marengo B. Longobardi M. Fazzi E. Orcesi S. Olivieri I. (2013). Inhibition of the de-myelinating properties of Aicardi-Goutières Syndrome lymphocytes by cathepsin D silencing. Biochem. Biophysical Res. Commun. 430 (3), 957–962. 10.1016/j.bbrc.2012.11.131 23261460

[B66] Rabe B. (2013). Aicardi–Goutières syndrome: clues from the RNase H2 knock-out mouse. J. Mol. Med. 91 (11), 1235–1240. 10.1007/s00109-013-1061-x 23744109

[B67] Rahimi Darehbagh R. Seyedoshohadaei S. A. Ramezani R. Rezaei N. (2024). Stem cell therapies for neurological disorders: current progress, challenges, and future perspectives. Eur. J. Med. Res. 29 (1), 386. 10.1186/s40001-024-01987-1 39054501 PMC11270957

[B68] Rehwinkel J. Mehdipour P. (2024). ADAR1: from basic mechanisms to inhibitors. Trends Cell Biol. 35, 59–73. 10.1016/j.tcb.2024.06.006 39030076 PMC11718369

[B69] Reichardt S. D. Amouret A. Muzzi C. Vettorazzi S. Tuckermann J. P. Lühder F. (2021). The role of glucocorticoids in inflammatory diseases. Cells 10, 2921. 10.3390/cells10112921 34831143 PMC8616489

[B70] Reijns M. A. M. Rabe B. Rigby R. E. Mill P. Astell K. R. Lettice L. A. (2012). Enzymatic removal of ribonucleotides from DNA is essential for mammalian genome integrity and development. Cell 149 (5), 1008–1022. 10.1016/j.cell.2012.04.011 22579044 PMC3383994

[B71] Rice G. I. Forte G. M. A. Szynkiewicz M. Chase D. S. Aeby A. Abdel-Hamid M. S. (2013). Assessment of interferon-related biomarkers in Aicardi-Goutières syndrome associated with mutations in TREX1, RNASEH2A, RNASEH2B, RNASEH2C, SAMHD1, and ADAR: a case-control study. Lancet. Neurology 12 (12), 1159–1169. 10.1016/S1474-4422(13)70258-8 24183309 PMC4349523

[B94] Sase S. Takanohashi A. Vanderver A. Almad A. (2018). Astrocytes, an active player in Aicardi-Goutières syndrome. Brain Pathol. 28 (3), 399–407. 10.1111/bpa.12600 29740948 PMC8028286

[B72] Schneider W. M. Chevillotte M. D. Rice C. M. (2014). Interferon-stimulated genes: a complex web of host defenses. Annu. Rev. Immunol. 32, 513–545. 10.1146/annurev-immunol-032713-120231 24555472 PMC4313732

[B73] Shi J. G. Chen X. Lee F. Emm T. Scherle P. A. Lo Y. (2014). The pharmacokinetics, pharmacodynamics, and safety of baricitinib, an oral JAK 1/2 inhibitor, in healthy volunteers. J. Clin. Pharmacol. 54 (12), 1354–1361. 10.1002/jcph.354 24965573

[B74] Shi J. G. Chen X. McGee R. F. Landman R. R. Emm T. Lo Y. (2011). The pharmacokinetics, pharmacodynamics, and safety of Orally dosed INCB018424 phosphate in healthy volunteers. J. Clin. Pharmacol. 51 (12), 1644–1654. 10.1177/0091270010389469 21257798

[B75] Strzelec M. Detka J. Mieszczak P. Sobocińska M. K. Majka M. (2023). Immunomodulation-a general review of the current state-of-the-art and new therapeutic strategies for targeting the immune system. Front. Immunol. 14, 1127704. 10.3389/fimmu.2023.1127704 36969193 PMC10033545

[B76] Sun L. Wu J. Du F. Chen X. Chen Z. J. (2013). Cyclic GMP-AMP synthase is a cytosolic DNA sensor that activates the type I interferon pathway. Science 339 (6121), 786–791. 10.1126/science.1232458 23258413 PMC3863629

[B77] Thomas C. A. Tejwani L. Trujillo C. A. Negraes P. D. Herai R. H. Mesci P. (2017). Modeling of TREX1-dependent autoimmune disease using human stem cells highlights L1 accumulation as a Source of neuroinflammation. Cell Stem Cell 21 (3), 319–331. 10.1016/j.stem.2017.07.009 28803918 PMC5591075

[B78] Tischner D. Reichardt H. M. (2007). Glucocorticoids in the control of neuroinflammation. Mol. Cell. Endocrinol. 275 (1), 62–70. 10.1016/j.mce.2007.03.007 17555867

[B79] Tonduti D. Fazzi E. Badolato R. Orcesi S. (2020). Novel and emerging treatments for Aicardi-Goutières syndrome. Expert Rev. Clin. Immunol. 16 (2), 189–198. 10.1080/1744666X.2019.1707663 31855085

[B80] Uggenti C. Lepelley A. Depp M. Badrock A. P. Rodero M. P. El-Daher M.-T. (2020). cGAS-mediated induction of type I interferon due to inborn errors of histone pre-mRNA processing. Nat. Genet. 52 (12), 1364–1372. 10.1038/s41588-020-00737-3 33230297

[B81] Vanderver A. Adang L. Gavazzi F. McDonald K. Helman G. Frank D. B. (2020). Janus kinase inhibition in the Aicardi-Goutières syndrome. N. Engl. J. Med. 383 (10), 986–989. 10.1056/NEJMc2001362 32877590 PMC7495410

[B82] Vanderver A. Prust M. Kadom N. Demarest S. Crow Y. J. Helman G. (2015). Early-onset Aicardi-Goutières syndrome: Magnetic Resonance imaging (MRI) pattern recognition. J. child neurology 30 (10), 1343–1348. 10.1177/0883073814562252 PMC4476968 25535058

[B92] Van Heteren J. T. Rozenberg F. Aronica E. Troost D. Lebon P. Kuijpers T. W. (2008). Astrocytes produce interferon-alpha and CXCL10, but not IL-6 or CXCL8, in Aicardi–Goutières syndrome. Glia 56 (5), 568–578. 10.1002/glia.20639 18240301

[B83] van Horssen J. van Schaik P. Witte M. (2019). Inflammation and mitochondrial dysfunction: a vicious circle in neurodegenerative disorders? Neurosci. Lett. 710, 132931. 10.1016/j.neulet.2017.06.050 28668382

[B84] Verstovsek S. Komrokji R. S. (2015). A comprehensive review of pacritinib in myelofibrosis. Future Oncol. 11 (20), 2819–2830. 10.2217/fon.15.200 26367195 PMC4918816

[B85] Viengkhou B. Hayashida E. McGlasson S. Emelianova K. Forbes D. Wiseman S. (2024a). The brain microvasculature is a primary mediator of interferon-α neurotoxicity in human cerebral interferonopathies. Immunity 57 (7), 1696–1709.e10. 10.1016/j.immuni.2024.05.017 38878770 PMC11250091

[B86] Viengkhou B. Hong C. Mazur C. Damle S. Gallo N. B. Fang T. C. (2024b). Interferon-α receptor antisense oligonucleotides reduce neuroinflammation and neuropathology in a mouse model of cerebral interferonopathy. J. Clin. Invest. 134 (4), e169562. 10.1172/JCI169562 38357922 PMC10869178

[B87] Volpi S. Picco P. Caorsi R. Candotti F. Gattorno M. (2016). Type I interferonopathies in pediatric rheumatology. Pediatr. Rheumatol. 14 (1), 35. 10.1186/s12969-016-0094-4 PMC4893274 27260006

[B88] Yan Y. Shin S. Jha B. S. Liu Q. Sheng J. Li F. (2013). Efficient and rapid Derivation of Primitive neural stem cells and generation of brain subtype neurons from human pluripotent stem cells. Stem Cells Transl. Med. 2 (12), 862–870. 10.5966/sctm.2013-0080 24113065 PMC3808201

[B89] Yasuda M. Nagappan-Chettiar S. Johnson-Venkatesh E. M. Umemori H. (2021). An activity-dependent determinant of synapse elimination in the mammalian brain. Neuron 109 (8), 1333–1349.e6. 10.1016/j.neuron.2021.03.006 33770504 PMC8068677

[B90] Yoshihara M. Hayashizaki Y. Murakawa Y. (2017). Genomic Instability of iPSCs: challenges towards their clinical applications. Stem Cell Rev. Rep. 13 (1), 7–16. 10.1007/s12015-016-9680-6 27592701 PMC5346115

[B91] Zhang S. Zheng R. Pan Y. Sun H. (2023). Potential therapeutic value of the STING inhibitors. Molecules 28 (7), 3127. 10.3390/molecules28073127 37049889 PMC10096477

