# Supplementary material for: Pharmacological evaluation of drug therapies in Aicardi-Goutières syndrome: insights from patient-derived neural stem cells
Source: Front Pharmacol. 2025 Mar 20;16:1549183. doi: 10.3389/fphar.2025.1549183 (PMC11966042; doi:10.3389/fphar.2025.1549183)
Supplement: Supplementary file 1 [file DataSheet1.pdf]

Pharmacological evaluation of drug therapies in Aicardi-Goutières syndrome: insights from patient-derived neural stem cells.

Stefania Braidotti [1], Rosalba Monica Ferraro [2,3], Raffaella Franca [4], Elena Genova [1], Francesco Giambuzzi [1], Andrea Mancini [5], Valentina Marinozzi [5], Letizia Pugnetti [1], Alessandra Tesser [1], Alberto Tommasini [1,4], Giuliana Decorti [1,4], Silvia Giliani [2,3] and Gabriele Stocco [1,4].

Supplementary Material

1 Mycoplasma detection in cell culture

iPSCs and NSCs were tested for *Mycoplasma* contamination once thawed. To check the presence of *Mycoplasma*, a polymerase chain reaction (PCR) method was used to assess the presence of a 16S rRNA gene (Young, Sung et al. 2010). Cells have always tested negative for *Mycoplasma*.

2 Supplementary Figures and Tables

2.1 Supplementary Figures

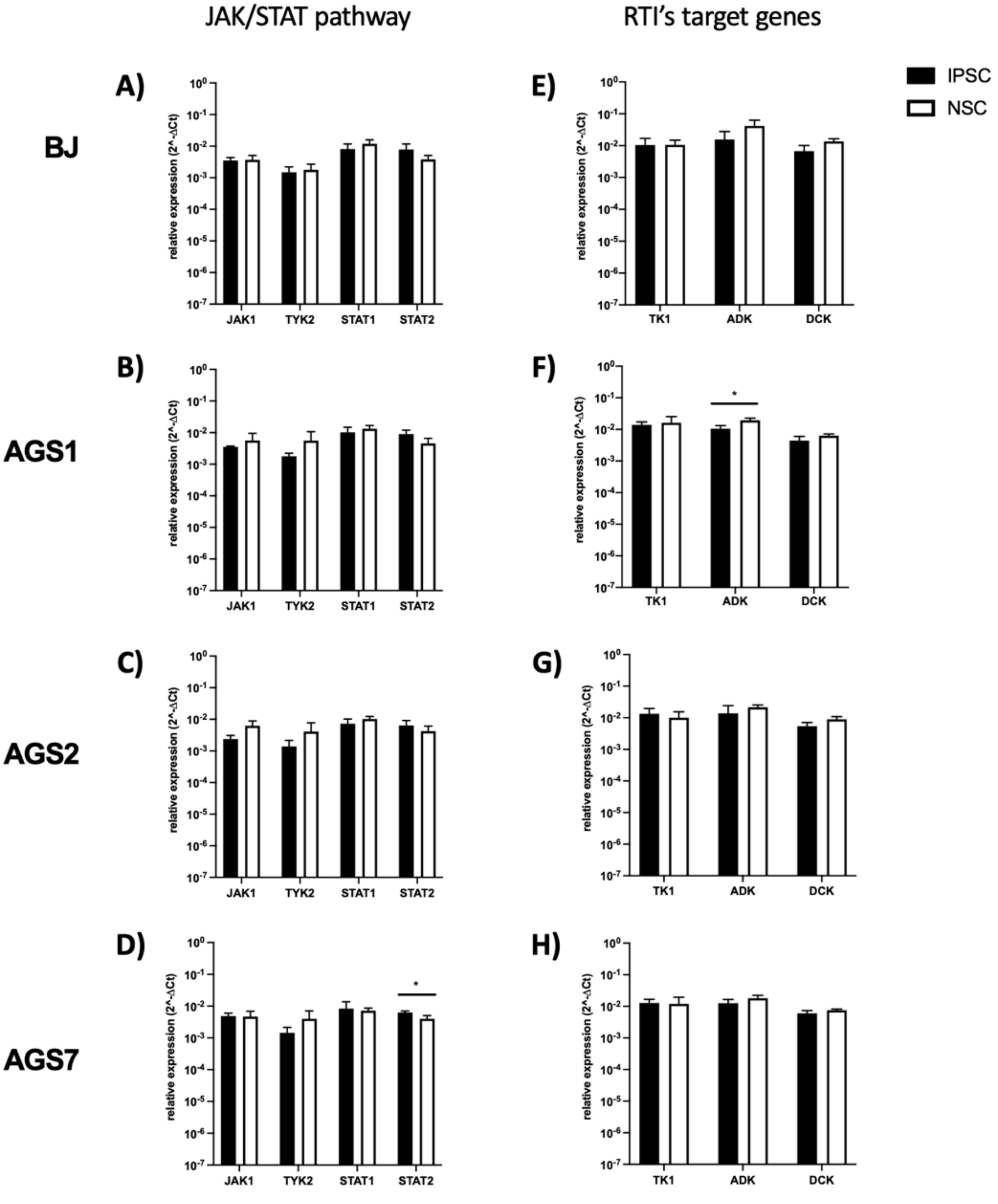

Supplementary Figure 1. Gene expression analysis of *JAK1*, *TYK2*, *STAT1*, *STAT2* and *TK1*, *ADK*, *DCK*. Data are normalized to housekeeping *β-actin* gene expression, and relative expression was calculated as $2^{-\Delta Ct}$. P-value according to t-test analysis, * $P<0.05$.

Supplementary Tables

DRUG	SOLVENT	STOCK SOLUTION (mM)	CONCENTRATION RANGE TESTED IN MTT ASSAY (IPSCs/NSCs)	Dilution Factor
Ruxolitinib	Ethanol 100%	32.6	$9.77x10^{-9}$ M – $2.0x10^{-5}$ M	1:3
Baricitinib	DMSO	50	$9.77x10^{-9}$ M – $2.0x10^{-5}$ M	1:3
Tofacitinib	DMSO	50	$9.77x10^{-9}$ M – $2.0x10^{-5}$ M	1:3
Pacritinib	DMSO	10	$7.0x10^{-9}$ M – $5.0x10^{-6}$ M	1:4
Lamivudine	H_2O	43.61	$9.77x10^{-9}$ M – $2.0x10^{-5}$ M	1:3
Abacavir Sulfate	H_2O	44.22	$9.77x10^{-9}$ M – $2.0x10^{-5}$ M	1:3
Zidovudine	H_2O	187.09	$9.77x10^{-9}$ M – $2.0x10^{-5}$ M	1:3
6-mercaptopurine	NaOH 0.1 M	200	$9.77x10^{-9}$ M – $2.0x10^{-5}$ M	1:3
Thioguanine	NaOH 0.1 M	200	$9.77x10^{-9}$ M – $2.0x10^{-5}$ M	1:3
Dexamethasone	0.9% NaCl	7.7	$7.77x10^{-6}$ M – $1.9x10^{-12}$ M	1:3

Supplementary Table 1. Drugs and their concentration range for *in vitro* drug sensitivity assays. In MTT assays, the percent solvent content is adjusted to the highest concentration used.

GENE	GENE FUNCTION	PRIMER	SEQUENCE 5'→3'
ACTB	House-keeping	Forward	CGCCGCCAGCTCACCATG
		Reverse	CACGATGGAGGGGAAGACGC
SOX2	IPSCs stemness gene	Forward	CCCAGCAGACTTCACATGT
		Reverse	CCTCCCATTTCCCTCGTTTT
OCT4	IPSCs stemness gene	Forward	CCTCACTTCACTGCACTGTA
		Reverse	CAGGTTTTCTTTCCCTAGCT
NES	NSCs stemness gene	Forward	ATGGAGACGTCGCTG
		Reverse	ACAGCCAGCTGGAAC
SOX1	NSCs stemness gene	Forward	TGCTTGTTCTGTTAACTCAC
		Reverse	AAAGAACCTCAGAGAGAGTC
PAX6	NSCs stemness gene	Forward	GAGTTTGAGAGAACCCATTATC
		Reverse	CATACCTGTATTCTTGCTTCAG
JAK1	Gene related to JAK/STAT signaling pathway	Forward	GAAAAACAAGATCCGGGAAG
		Reverse	TCCATTTTCTTGTTGTCCTG
TYK2		Forward	CTCCTTGCTTCAATCTCTTTG

	Gene related to JAK/STAT signaling pathway	Reverse	ACCTTATGCGGAAATATAGC
STAT1	Gene related to JAK/STAT signaling pathway	Forward	ACCCAATCCAGATGTCTATG
		Reverse	GAGCCTGATTAATCTCTGG
STAT2	Gene related to JAK/STAT signaling pathway	Forward	ATATAAGATCCAGGCCAAAGG
		Reverse	CAGTAGCTCGATTAGGGTAG
TK1	Zidovudine target gene	Forward	AAAAGCACAGAGTTGATGAG
		Reverse	GAGTGTCTTTGGCATACTTG
ADK	Abacavir target gene	Forward	CCAAAGATGAACTCAAAGAGG
		Reverse	AGAAAACCTCCAACAAATGC
DCK	Lamivudine drugs target gene	Forward	GAGGAACTTACAATGTCTCAG
		Reverse	TGTTTGGAAGGTAAAAGACC
HPRT1	Thiopurine drugs target gene	Forward	ATAAGCCAGACTTTGTTGG
		Reverse	ATAGGACTCCAGATGTTTCC
NR3C1	Glucocorticoids drugs target gene	Forward	ACTGCTTCTCTCTTCAGTTC
		Reverse	GATTTTCAAACCACTTCATGC

Supplementary Table 2. Primer sequences (Sigma-Aldrich, Italy) for real-time PCR analysis of IPSCs stemness genes, NSCs stemness genes, genes involved in the activation of type I IFN and of antiretroviral drug pathways. *ACTB*, beta-actin; *ADK*, adenosine kinase; *DCK*, Deoxycytidine Kinase; *HPRT1*, hypoxanthine phosphoribosyltransferase 1; *JAK1*, Janus kinase 1; *NES*, Nestin; *NR3C1*, nuclear receptor subfamily 3 group C member 1; *OCT4*, POU class 5 homeobox 1; *PAX6*, paired type homeobox 6; *SOX2*, SRY-box 2; *STAT1*, Signal transducer and activator of transcription 1; *STAT2*, Signal transducer and activator of transcription 2; *TK1*, thymidine kinase 1; *TYK2*, Tyrosine kinase 2.

References

Young, L., J. Sung, G. Stacey and J. R. Masters (2010). "Detection of Mycoplasma in cell cultures." Nature Protocols **5**(5): 929-934.
